# Tyrosine phosphorylation of the scaffold protein IQGAP1 in the MET pathway alters function

**DOI:** 10.1074/jbc.RA120.015891

**Published:** 2025-01-13

**Authors:** Andrew C. Hedman, Dean E. McNulty, Zhigang Li, Laëtitia Gorisse, Roland S. Annan, David B. Sacks

**Affiliations:** 1 Department of Laboratory Medicine, National Institutes of Health, Bethesda, Maryland, USA; 2 Discovery Analytical, GlaxoSmithKline, Collegeville, Pennsylvania, USA

**Keywords:** receptor tyrosine kinase, phosphorylation, scaffold protein, protein phosphorylation, mass spectrometry (MS), phosphotyrosine signaling, IQGAP1, receptor tyrosine-kinase, phosphotyrosine, IQGAP2

## Abstract

IQGAP1 is a key scaffold protein that regulates numerous cellular processes and signaling pathways. Analogous to many other cellular proteins, IQGAP1 undergoes post-translational modifications, including phosphorylation. Nevertheless, very little is known about the specific sites of phosphorylation or the effects on IQGAP1 function. Here, using several approaches, including MS, site-directed mutagenesis, siRNA-mediated gene silencing, and chemical inhibitors, we identified the specific tyrosine residues that are phosphorylated on IQGAP1 and evaluated the effect on function. Tyr-172, Tyr-654, Tyr-855, and Tyr-1510 were phosphorylated on IQGAP1 when phosphotyrosine phosphatase activity was inhibited in cells. IQGAP1 was phosphorylated exclusively on Tyr-1510 under conditions with enhanced MET or c-Src signaling, including in human lung cancer cell lines. This phosphorylation was significantly reduced by chemical inhibitors of MET or c-Src or by siRNA-mediated knockdown of MET. To investigate the biological sequelae of phosphorylation, we generated a nonphosphorylatable IQGAP1 construct by replacing Tyr-1510 with alanine. The ability of hepatocyte growth factor, the ligand for MET, to promote AKT activation and cell migration was significantly greater when IQGAP1-null cells were reconstituted with IQGAP1 Y1510A than when cells were reconstituted with WT IQGAP1. Collectively, our data suggest that phosphorylation of Tyr-1510 of IQGAP1 alters cell function. Because increased MET signaling is implicated in the development and progression of several types of carcinoma, IQGAP1 may be a potential therapeutic target in selected malignancies.

IQGAP1 is a protein scaffold that participates in signaling pathways, such as the mitogen-activated protein kinase (MAPK) (1, 2) and phosphoinositide-3-kinase (PI3K)/AKT (3, 4) pathways, and modulates the cytoskeleton via the small GTPases, including CDC42 and RAC1 (5, 6). Investigations have identified over 100 protein interactors for IQGAP1 (7, 8). In humans, there are two IQGAP1-related proteins, IQGAP2 and IQGAP3, each of which has its own unique roles but shares many functions with IQGAP1 (7). Whereas cellular proteins are regulated by numerous post-translational modifications (*e.g.* phosphorylation, ubiquitination, acetylation, SUMOylation, or ISGylation), the modulation of IQGAP1 by these modifications has received limited characterization. The major reported functional phosphorylation sites in IQGAP1 are Ser-1441/Ser-1443 which are catalyzed by the protein kinase C (PKC) isoforms PKCα (9) or PKCε (10). Phosphorylation of these amino acid residues promotes neurite outgrowth (10). Proteomic studies by MS have identified several amino acids, including some tyrosine residues, that are phosphorylated in IQGAP1 (11), but most have no function assigned to them. A few biochemical studies have detected phosphotyrosine bands by Western blotting after IQGAP1 immunoprecipitation. For example, vascular endothelial growth factor (VEGF) stimulates tyrosine phosphorylation of IQGAP1 (12, 13) via c-Src (14). In addition, platelet-derived growth factor (15) and colony-stimulating factor-1 (CSF-1) (14) have been reported to promote IQGAP1 tyrosine phosphorylation in cells. However, in none of these cases was the specific phosphorylation sites identified. By contrast, MS analysis did not detect any tyrosine phosphorylation of IQGAP1 in cells stimulated with epidermal growth factor (EGF) (9). Rather, EGF stimulates Ser-1441/1443 phosphorylation of IQGAP1 through PKCα.

MET is the receptor tyrosine kinase for hepatocyte growth factor (HGF) (16). Binding of HGF induces dimerization and autophosphorylation of MET, which then initiates signaling cascades to activate intracellular signaling pathways, such as the MAPK pathway, the PI3K/AKT pathway, and c-Src (16). This results in cell proliferation, motility, wound healing, cell cycle progression, and protection from apoptosis (17, 18). MET has been the focus of a large body of research because dysregulation of MET signaling is implicated in cancer progression and metastasis (19). Interestingly, IQGAP1 participates in some functions of HGF signaling. HGF is known to enhance barrier functions of endothelial cells by regulating cytoskeletal organization (20). IQGAP1 has a role in this process as HGF promotes IQGAP1 association with EB-1 (a microtubule-binding protein), cortactin (an actin-binding protein), and ASEF1 (a Rac-GEF) (21, 22) to enhance barrier functions. Moreover, HGF increases the association of IQGAP1 with the Ser/Thr-protein kinase PAK6, which enhances PAK6 activity and promotes the disassembly of cell-cell junctions (23). Whereas these studies used cells stimulated with HGF, a possible link between IQGAP1 and MET has not been clearly established.

Site-specific phosphorylation of proteins is a fundamental and common post-translational modification that regulates intracellular signaling (24). Phosphoproteomic databases derived from MS analyses contain >100,000 annotated phosphosites, but kinases have been identified for less than 5% of these, and knowledge of function is negligible (25). Moreover, almost half the phosphorylated sites in data sets may be false positives (26). No published study has confirmed the sites of tyrosine phosphorylation on IQGAP1 or identified the specific kinases that catalyze the phosphorylation. Furthermore, functional consequences of IQGAP1 tyrosine phosphorylation have not been determined. Therefore, in this study, we specifically investigated tyrosine phosphorylation of IQGAP1. We identified by MS several tyrosine residues that are phosphorylated on IQGAP1 in mammalian cells, elucidated stimuli that promote this phosphorylation, and examined functional consequences of the post-translational modification.

## Results

### IQGAP1 is tyrosine-phosphorylated in cells

**Figure 1:**
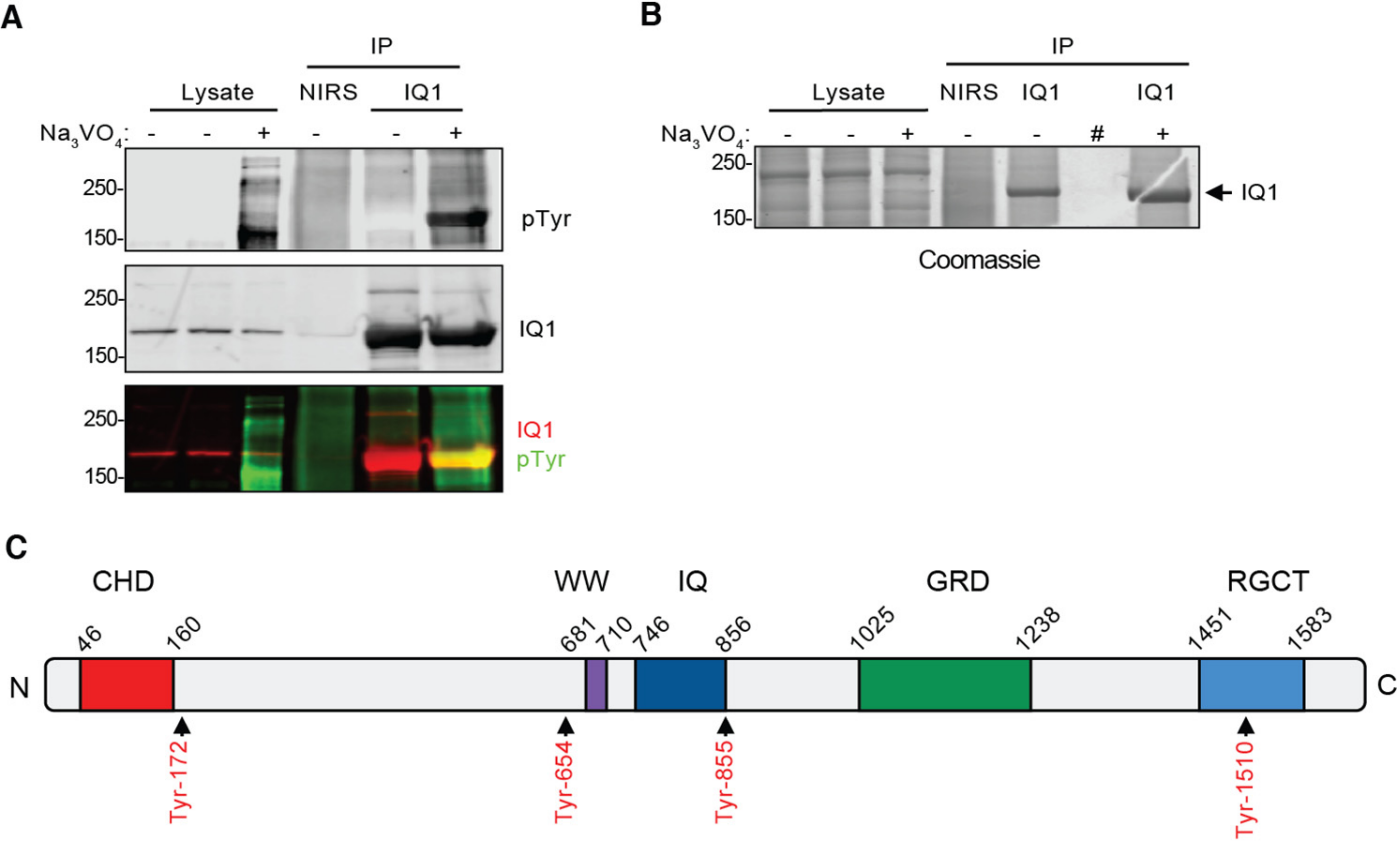
**IQGAP1 is tyrosine-phosphorylated in cells incubated with vanadate.***A*, MDA-MB-231 cells were treated with (+) or without (−) 2 mm Na_3_VO_4_ for 16 h. Endogenous IQGAP1 was immunoprecipitated (IP) with anti-IQGAP1 (*IQ1*) antibody from cell lysates. NIRS was used as a control. Lysate and IP samples were processed by Western blotting and probed with antibodies to phosphotyrosine (pTyr; *top*) and IQGAP1 (*middle*). The overlap between IQGAP1 (*red*) and pTyr (*green*) signals is visible in the *merged image* (*bottom*). The position of migration of molecular mass markers (in kDa) is depicted on the *left* of the blots. Data are representative of three independent experiments. *B*, MDA-MB-231 cells were treated for 16 h with (+) or without (−) Na_3_VO_4_, and IQGAP1 was IP. NIRS was used as an immunoprecipitation control. #, empty lane. Samples were resolved by SDS-PAGE, and the gel was stained with Colloidal Coomassie. IQGAP1 bands (*arrow*) were excised from the gel and analyzed by MS. *C*, schematic of tyrosine phosphorylation sites on IQGAP1 identified by MS. IQGAP1 contains five domains: a calponin-homology domain (*CHD*), a domain containing two tryptophan residues (*WW*), an IQ domain (*IQ*), a GAP-related domain (*GRD*), and a RasGAP_C-terminus (*RGCT*). The phosphorylated tyrosine residues (*red*) are labeled.

Whereas a number of global phosphoproteomic analyses by MS have detected tyrosine phosphorylation of IQGAP1 (11), under basal experimental conditions, tyrosine phosphorylation of IQGAP1 in many cell lines cultured in serum is minimal (9, 27) (Fig. 1*A*). Some publications have shown phosphotyrosine bands that co-migrate with immunoprecipitated IQGAP1 and are detectable by Western blotting (13, 14, 28, 29). Nevertheless, these tyrosine phosphorylation sites have not been validated to be on IQGAP1. To observe robust reproducible tyrosine phosphorylation of IQGAP1, we used sodium orthovanadate (Na_3_VO_4_). This cell-permeable compound inhibits phosphotyrosine phosphatase activity (30, 31), thereby enhancing the detection of tyrosine phosphorylation of numerous cellular proteins. We used MDA-MB-231 cells, as these contain high levels of IQGAP1 (32). MDA-MB-231 cells were incubated with Na_3_VO_4_ for 16 h and lysed, and endogenous IQGAP1 was immunoprecipitated. Samples were analyzed by SDS-PAGE, and Western blots were probed with anti-IQGAP1 and anti-phosphotyrosine antibodies. As anticipated, vanadate enables detection of tyrosine phosphorylation of multiple proteins in the cell lysates (Fig. 1*A* (*top*), lysate). No tyrosine phosphorylation of immunoprecipitated IQGAP1 was detected in control cells. By contrast, clear tyrosine phosphorylation of endogenous IQGAP1 was visible in the presence of vanadate, as demonstrated by co-migration of IQGAP1 (*red*) and phosphotyrosine (*green*) bands (Fig. 1*A*, *bottom*). No IQGAP1 was detected in the samples precipitated with nonimmune rabbit serum (NIRS), verifying the specificity of the of the immunoprecipitation for IQGAP1. Inhibition of phosphotyrosine phosphatases with vanadate enabled the clear detection of tyrosine phosphorylation of endogenous IQGAP1 protein. These findings suggest that IQGAP1 is tyrosine-phosphorylated in cells, but under tight control by tyrosine phosphatases.

To identify the specific tyrosine residue(s) that are phosphorylated, we immunoprecipitated endogenous IQGAP1 from vanadate- and control-treated MDA-MB-231 cells and analyzed the appropriate SDS-PAGE bands (Fig. 1*B*) by MS. Four tyrosine-phosphorylated residues were detected on IQGAP1: Tyr-172, Tyr-654, Tyr-855, and Tyr-1510 (Fig. 1*C* and Figs. S1 and S2).

In addition to identifying tyrosine phosphorylation sites on IQGAP1, we performed similar studies with another member of the IQGAP protein family, IQGAP2. To ascertain whether endogenous IQGAP2 is also phosphorylated on tyrosine, we used HepG2 cells because IQGAP2 is expressed predominantly in the liver (8). HepG2 cells were incubated with vanadate or control, IQGAP2 was immunoprecipitated, and samples were analyzed by Western blotting and MS as described above. Analogous to the findings with IQGAP1, clear tyrosine phosphorylation of IQGAP2 was observed by Western blotting in the presence, but not the absence, of vanadate (Fig. S3A). Furthermore, we identified three tyrosine-phosphorylated residues on IQGAP2: Tyr-14, Tyr-93, and Tyr-770 (Figs. S3C, S4, and S5). The only tyrosine-phosphorylated residue that is conserved between the two proteins is Tyr-855 in IQGAP1, which corresponds to Tyr-770 in IQGAP2.

### MET stimulates IQGAP1 tyrosine phosphorylation

**Figure 2:**
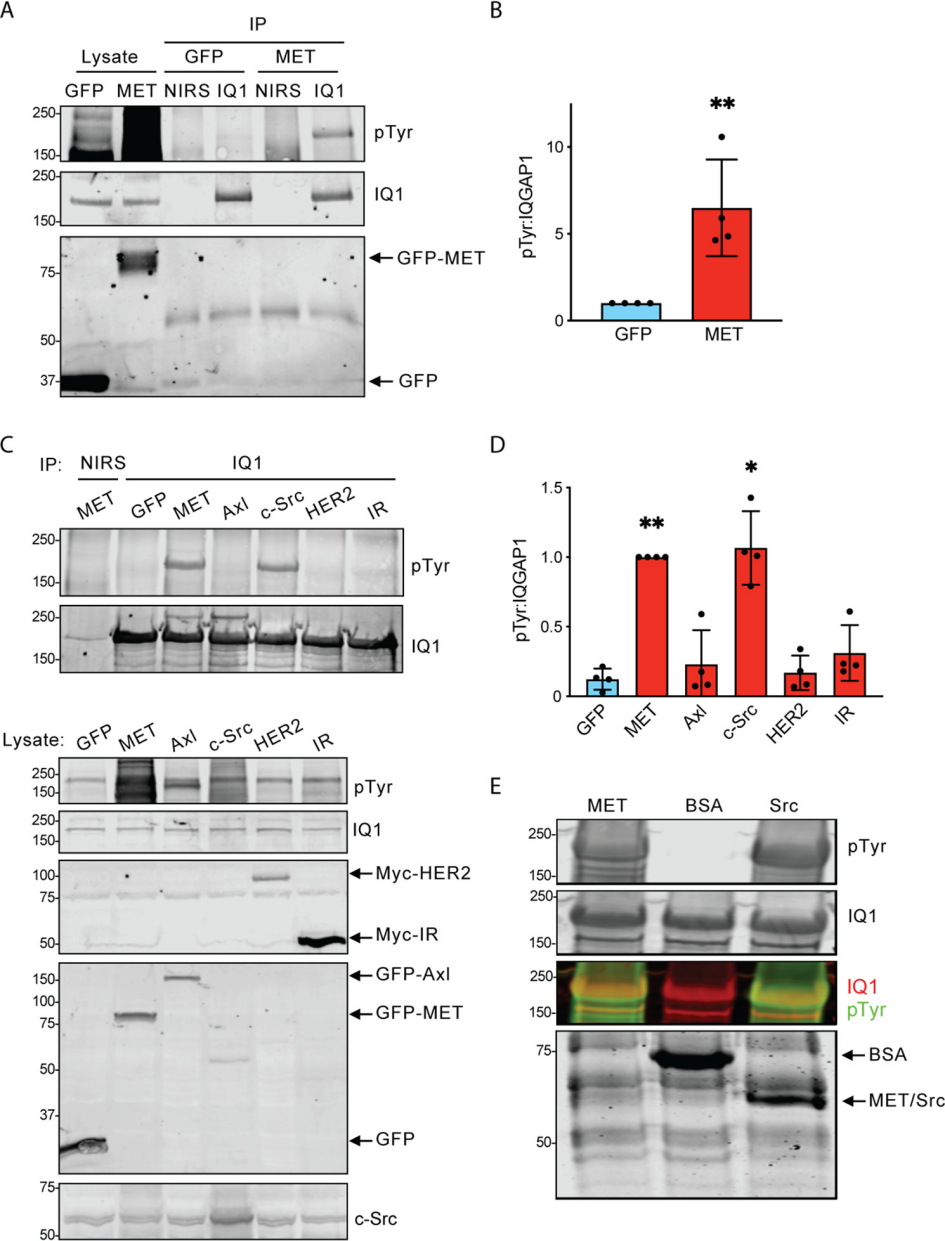
**MET and c-Src stimulate IQGAP1 tyrosine phosphorylation.***A*, HEK293 cells were transfected with GFP or the GFP-tagged cytoplasmic domain of MET (MET, amino acids 954–1390). Endogenous IQGAP1 was IP with anti-IQGAP1 (*IQ1*) antibodies. NIRS was used as a control for immunoprecipitation. Lysates not subjected to immunoprecipitation were processed in parallel. Samples were analyzed by Western blotting and probed with antibodies to pTyr and IQGAP1 (*top* and *middle*, respectively). The *bottom panel* was probed with anti-GFP antibody to show the expression of GFP and GFP-MET. B, the pTyr and IQGAP1 bands were quantified using LI-COR Image Studio. Graphs depict the ratio of pTyr to IQGAP1, with the GFP-transfected condition set to 1. Data are means ± S.D. (*error bars*) (*n* = 4). **, *p* < 0.01 by Student's *t* test. *C*, HEK293 cells were transfected with plasmids for GFP, GFP-tagged MET (cytoplasmic domain), GFP-Axl, untagged c-Src, Myc-tagged HER2, or Myc-IR (cytoplasmic domain of the insulin receptor). 48 h after transfection, cells were lysed, and IQGAP1 was IP. NIRS was used as a control for immunoprecipitation. 2% of the cell lysate were run on a separate gel to demonstrate protein expression. Samples were analyzed by SDS-PAGE, and Western blots were probed with antibodies to pTyr, IQGAP1, Myc, GFP, or c-Src. Data are representative of four independent experiments. *D*, the amount of pTyr that co-migrates with immunoprecipitated IQGAP1 was quantified with LI-COR Image Studio. Data were corrected for the amount of immunoprecipitated IQGAP1 in the corresponding sample. Values were normalized to the amount of phosphotyrosine in cells transfected with MET. Data are means ± S.D. (*n* = 4). *, *p* < 0.05; **, *p* < 0.01 by one-way ANOVA compared with cells transfected with GFP alone. *E*, purified recombinant GST-IQGAP1 was incubated with pure MET, BSA, or c-Src in an *in vitro* phosphorylation assay. Samples were resolved by SDS-PAGE, and the gel was cut at ∼100 kDa. The top portion was transferred to PVDF membranes, which were probed with antibodies to pTyr and IQGAP1 (*top* and *second panels*, respectively). The overlap between IQGAP1 (*red*) and pTyr (*green*) signals is visible in the merged image (*third panel*). The lower portion of the gel was stained with Coomassie Blue to visualize the added proteins (*bottom panel*). The *lower* molecular bands visible in the *bottom panel* are probably degradation products of IQGAP1.

To identify potential kinases that mediate tyrosine phosphorylation of IQGAP1, we transfected HEK293 cells with selected tyrosine kinases. The GFP-tagged cytoplasmic domain of MET (GFP-MET_cyto_, amino acids 954–1390) or GFP alone was expressed. Endogenous IQGAP1 was immunoprecipitated and analyzed by Western blotting. Tyrosine phosphorylation of IQGAP1 is minimal in cells transfected with GFP alone (Fig. 2*A*). Expression of GFP-MET_cyto_ stimulates IQGAP1 tyrosine phosphorylation (Fig. 2*A*). Quantification of the ratio of phosphotyrosine to IQGAP1 revealed a significant increase (6.49 ± 2.77-fold, mean ± S.D.) in IQGAP1 tyrosine phosphorylation in cells transfected with GFP-MET_cyto_ compared with cells transfected with GFP alone (Fig. 2*B*). In addition, we transfected cells with either MET_cyto_, Axl (33), c-Src (14), HER2 (34), or the cytoplasmic domain of the insulin receptor (IR_cyto_) (27). Neither Axl, HER2, nor IR_cyto_ induced substantial tyrosine phosphorylation of IQGAP1 (Fig. 2*C*). In contrast, c-Src increased tyrosine phosphorylation of IQGAP1 by approximately the same magnitude as MET_cyto_ (Fig. 2, *C* and *D*). Overexpression of the kinases was confirmed with antibodies to GFP, Myc, and c-Src (Fig. 2*C*).

To examine whether MET or c-Src phosphorylates IQGAP1 directly, we performed an *in vitro* protein phosphorylation assay. GST-IQGAP1, purified from *Escherichia coli,* was incubated with purified recombinant MET or c-Src. Samples were analyzed by SDS-PAGE and Western blotting with antibodies to phosphotyrosine and IQGAP1. IQGAP1 was phosphorylated on tyrosine by MET or c-Src (Fig. 2*E*). As anticipated, no phosphotyrosine was detected when IQGAP1 was incubated with BSA. Because the kinases are not 100% pure, we cannot completely exclude the possibility that a contaminating kinase contributed to the phosphorylation. Nevertheless, our data strongly suggest that MET and c-Src directly catalyze the phosphorylation of IQGAP1 *in vitro*.

**Figure 3:**
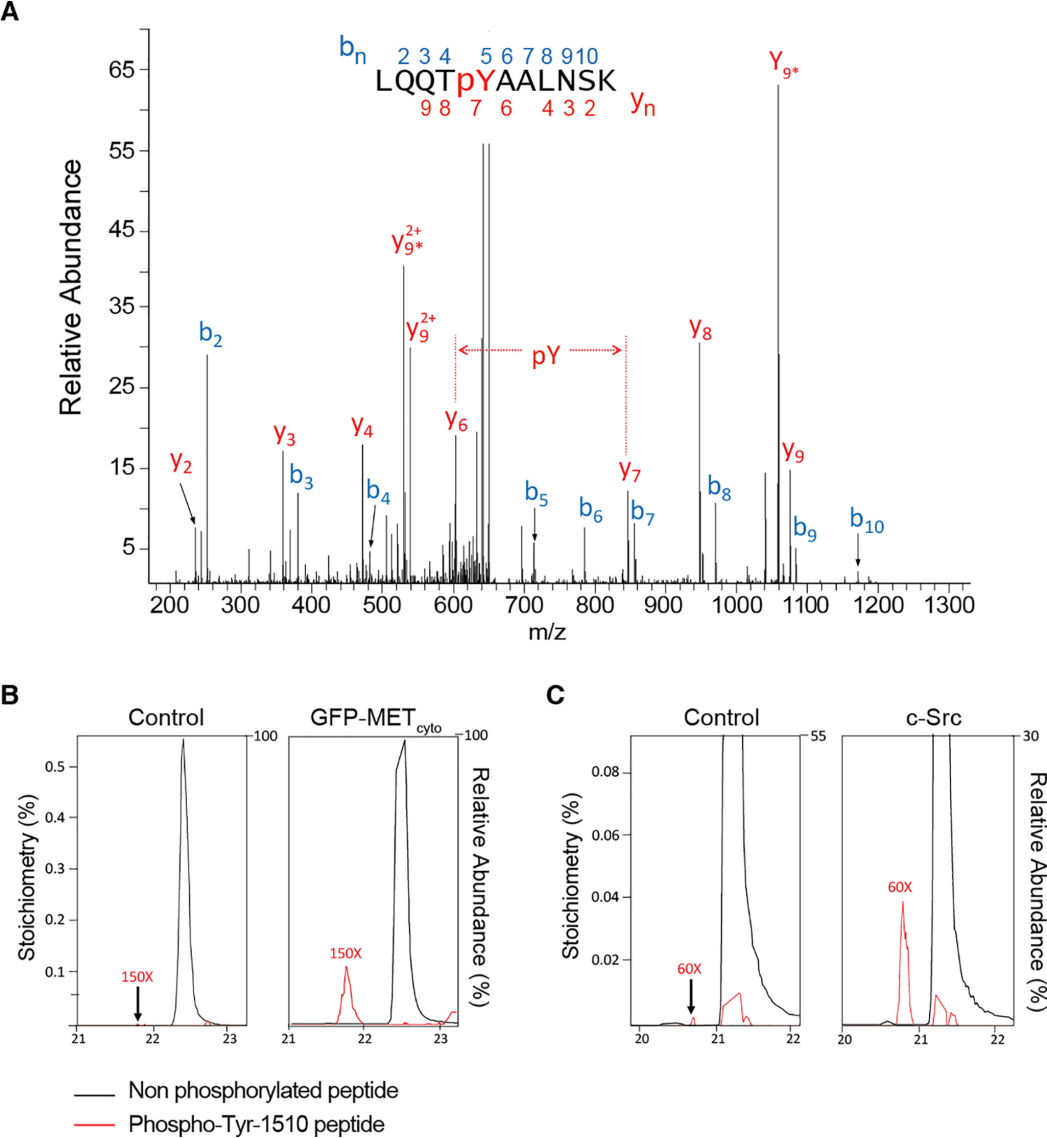
**Phosphorylation of IQGAP1 Tyr-1510.***A*, LC–MS/MS spectrum of the [M + 2H]^2+^ ion at *m*/*z* 658.8173 for the phosphorylated IQGAP1 tryptic peptide LQQTpYAALNSK (amino acids 1506–1516). The mass difference between the threonine at position 4 and the alanine at position 6 corresponds to phosphotyrosine. Sequence-specific fragment ions are labeled according to Biemann. Details are shown in Fig. S2D. *B* and *C*, transfection of HEK293 cells with plasmids expressing GFP-MET_cyto_ (*B*) or c-Src (*C*) increases phosphorylation on Tyr-1510 relative to treatment with control; shown are extracted ion chromatograms for the tryptic peptide LQQTYAALNSK with (*red*) and without (*black*) phosphorylation on Tyr-1510. Chromatograms were normalized where necessary to account for unequal loadings between samples. The signal for phosphorylated peptide was magnified in each case to visualize the change in stoichiometry.

To determine which tyrosine residues are influenced by MET and c-Src expression, we immunoprecipitated IQGAP1 from HEK-293 cells transfected with either MET_cyto_, c-Src, or control vectors (as in Fig. 2, *A* and *C*) and analyzed the appropriate SDS-PAGE bands by MS. Experiments were performed without incubating cells with vanadate to control intracellular phosphotyrosine phosphatase activity. MS analysis revealed that phosphorylation occurred only on Tyr-1510 (Fig. 3*A*) on IQGAP1 in cells that overexpress either MET_cyto_ or c-Src (Fig. 3*B* and Fig. S6). By contrast, no tyrosine phosphorylation of IQGAP1 was detectable in cells transfected with empty vector.

Whereas a few studies have examined the regulation of IQGAP1 by HGF (21, 22, 23), no publication has studied phosphorylation of IQGAP1 by MET. Therefore, we decided to investigate the role of MET in IQGAP1 phosphorylation.

### IQGAP1 is constitutively tyrosine-phosphorylated in NSCLC cell lines with MET up-regulation

**Figure 4:**
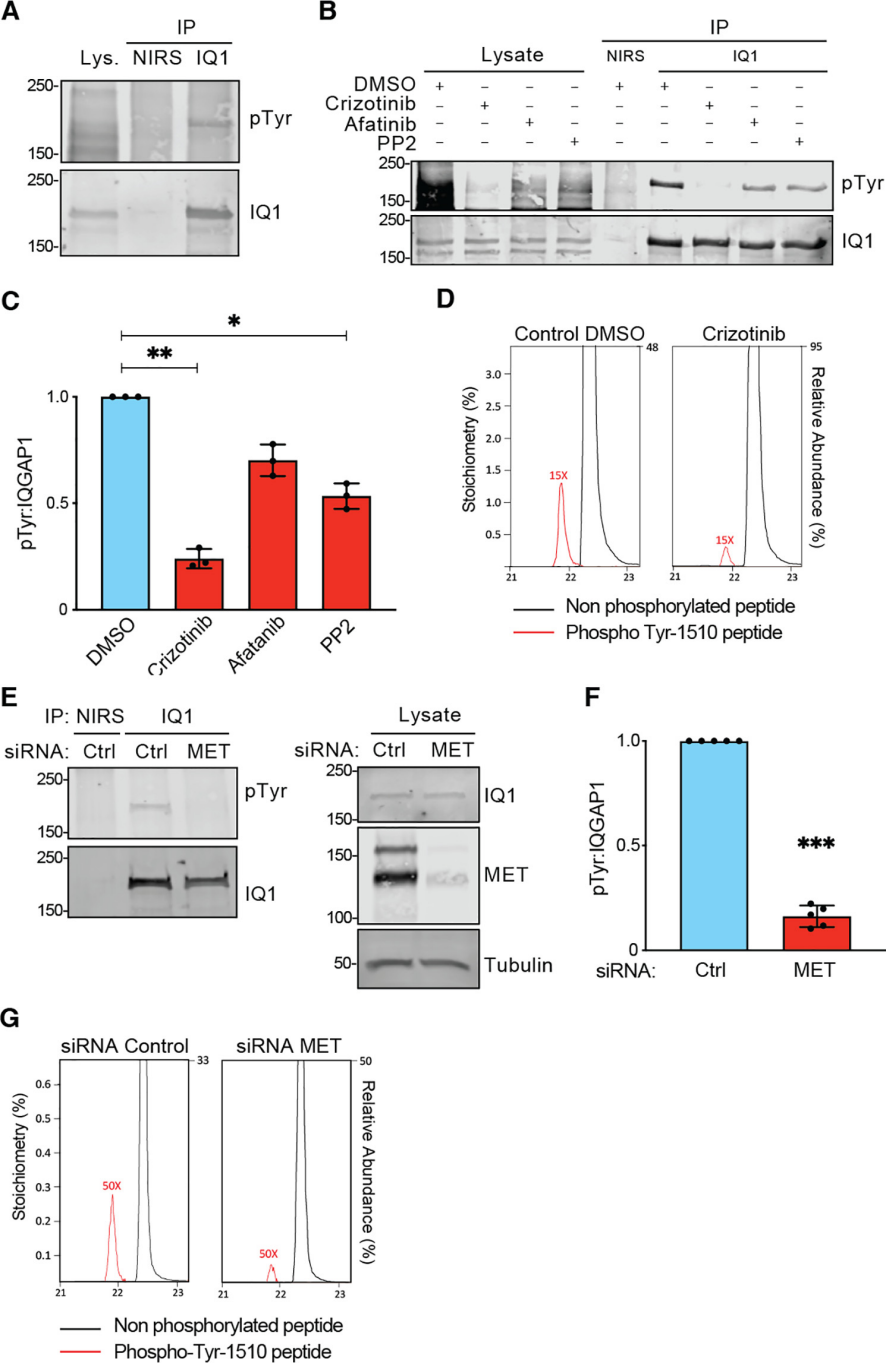
**IQGAP1 is tyrosine phosphorylated in H1993 cells.***A*, endogenous IQGAP1 was IP from H1993 cells with anti-IQGAP1 antibodies (*IQ1*). NIRS was used as a control. Samples were analyzed by Western blotting with antibodies to pTyr and IQGAP1. Data are representative of three independent experiments. 2% of the immunoprecipitation lysate was loaded separately (*Lys*.). *B*, H1993 cells were cultured for 24 h with DMSO (vehicle), 100 nm crizotinib (MET inhibitor), 5 μm afatanib (EGFR inhibitor), or 5 μm PP2 (c-Src inhibitor). Then IQGAP1 was IP with anti-IQGAP1 antibodies. NIRS served as a control. Samples were analyzed by Western blotting with antibodies to pTyr and IQGAP1. Data are representative of three independent experiments. *C*, the amount of pTyr that co-migrates with immunoprecipitated IQGAP1 was quantified with LI-COR Image Studio. The ratio of pTyr to IQGAP1 was graphed, and data were normalized to samples treated with DMSO. Graphs depict means ± S.D. (*error bars*) (*n* = 3). *, *p* < 0.05; **, *p* < 0.01 by one-way ANOVA. *D*, treatment of H1993 cells with crizotinib (*right*) decreases phosphorylation on Tyr-1510 relative to treatment with DMSO (*left*); extracted ion chromatograms are shown for the tryptic peptide LQQTYAALNSK with (*red*) and without (*black*) phosphorylation on Tyr-1510. Chromatograms were normalized where necessary to account for unequal loadings between samples. The signal for phosphorylated peptide was magnified in each case to visualize the change in stoichiometry. *E*, H1993 cells were transfected with control (*Ctrl*) siRNA or siRNA directed against MET. 48 h after transfection, endogenous IQGAP1 was IP. NIRS was used as a control. Samples were analyzed by Western blotting with antibodies to pTyr and IQGAP1 (*IQ1*) (*left panels*). Equal amounts of protein lysate were processed by Western blotting with antibodies to IQGAP1, MET, and tubulin (loading control) (*right panels*). Data are representative of five independent experiments. *F*, the graph depicts the ratio of pTyr to immunoprecipitated IQGAP1 as quantified by LI-COR Image Studio. Ratios are normalized to the control siRNA condition. Data are means ± S.D. (*n* = 5). ***, *p* < 0.001 by Student's *t* test. *G*, transfection of H1993 cells with siRNA targeting MET (*right*) decreases phosphorylation on Tyr-1510 relative to transfection with control siRNA (*left*); extracted ion chromatograms for the tryptic peptide LQQTYAALNSK with (*red*) and without (*black*) phosphorylation on Tyr-1510. Chromatograms were normalized where necessary to account for unequal loadings between samples. The signal for phosphorylated peptide was magnified in each case to visualize the change in stoichiometry.

IQGAP1 displays minimal or undetectable basal phosphorylation in the cell lines we examined, namely MDA-MB-231 (Fig. 1*A*) and HEK293 (Fig. 2*A*) cells. However, overexpression of MET in the latter cell line results in robust phosphorylation of IQGAP1 on Tyr-1510 (Figure 2, Figure 3). Therefore, we assessed phosphorylation of endogenous IQGAP1 in non-small cell lung carcinoma (NSCLC) cell lines where MET signaling is up-regulated (35). H1993 cells express high levels of constitutively active MET (35). Endogenous IQGAP1, immunoprecipitated from H1993 cells, exhibits clear tyrosine phosphorylation (Fig. 4*A*). To determine whether IQGAP1 tyrosine phosphorylation is mediated by MET in these cells, we used crizotinib, an established MET inhibitor (36, 37). Crizotinib significantly decreased (by 82.0%) tyrosine phosphorylation of IQGAP1 in H1993 cells (Fig. 4, *B* and *C*). Active MET can also promote activation of the EGF receptor (EGFR) and c-Src tyrosine kinase (38). Therefore, we inhibited the EGFR or c-Src with afatanib or PP2, respectively. Afatanib and PP2 attenuated IQGAP1 tyrosine phosphorylation in H1993 cells by 35.1 and 52.8%, respectively, but the magnitude of reduction was considerably less than that produced by the MET inhibitor crizotinib (Fig. 4, *B* and *C*).

To confirm that inhibition of MET activity by crizotinib influenced MET-dependent IQGAP1 phosphorylation, we analyzed the crizotinib-treated H1993 cells by MS. We again found that only Tyr-1510 was phosphorylated and that phosphorylation at this site was reduced almost 4-fold following MET inhibition (Fig. 4*D* and Fig. S6).

To unequivocally establish that MET was responsible for inducing IQGAP1 phosphorylation, we knocked down MET with siRNA. H1993 cells were transfected with siRNA targeting MET or control sequences. siRNA reduced MET expression (Fig. 4*E*, *right*). Under these conditions, tyrosine phosphorylation of IQGAP1 was significantly decreased by 83.7 ± 5.1% (mean ± S.D.) (Fig. 4, *E* and *F*). Analysis of IQGAP1 in these cells by MS again confirmed reduction in phosphorylation by ∼4-fold at Tyr-1510 (Fig. 4*G* and Fig. S6), consistent with the Western blotting data.

**Figure 5:**
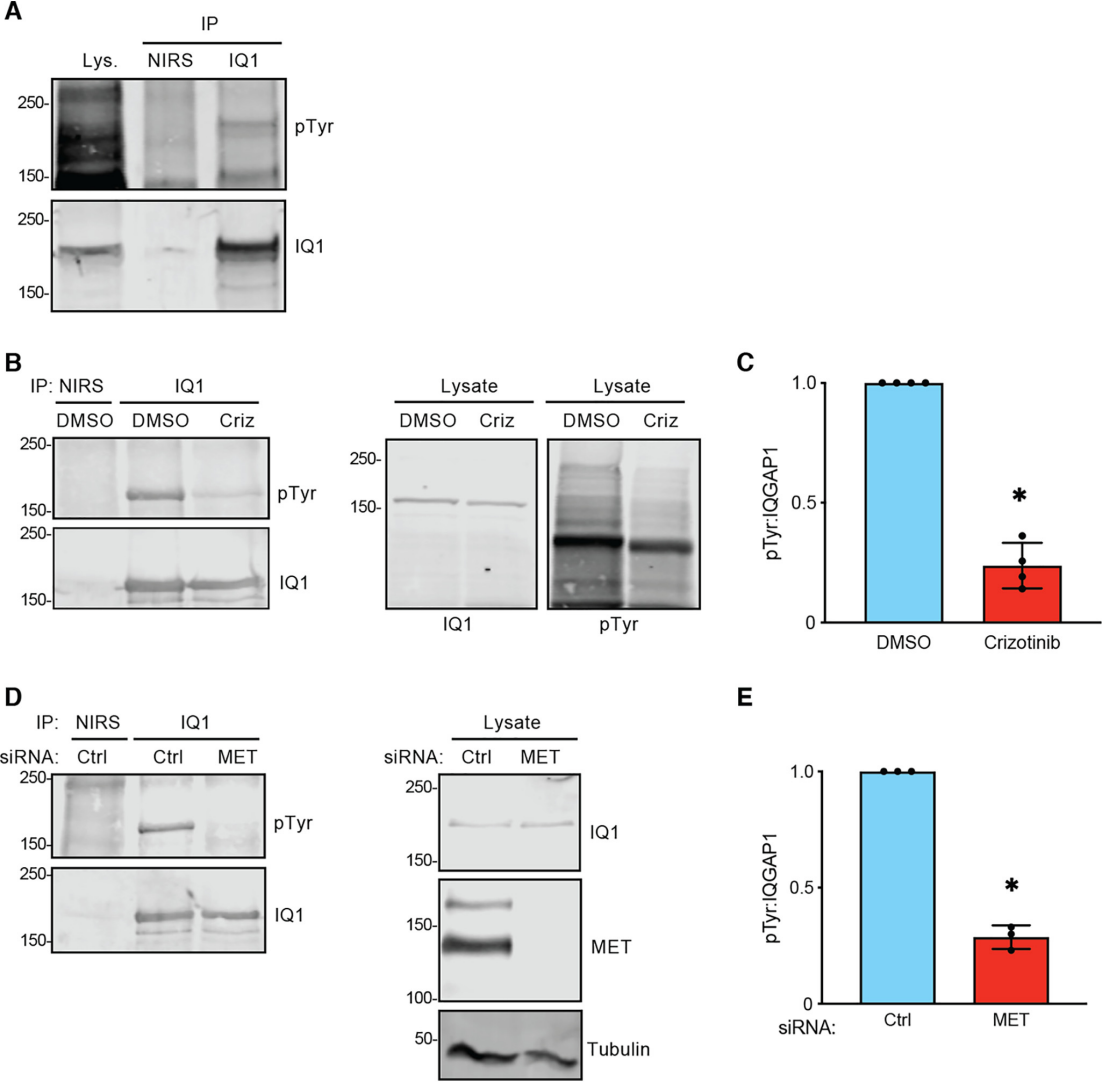
**IQGAP1 is tyrosine-phosphorylated in EBC-1 cells.***A*, endogenous IQGAP1 was IP from EBC-1 cells with anti-IQGAP1 (*IQ1*) antibodies. NIRS was used as a control. Samples were analyzed by Western blotting with antibodies to pTyr and IQGAP1. Data are representative of three independent experiments. *B*, EBC-1 cells were cultured for 24 h with DMSO (vehicle control) or 100 nm crizotinib (*Criz*). Then IQGAP1 was IP with anti-IQGAP1 antibodies. NIRS was a control. Samples were analyzed by Western blotting with antibodies to pTyr and IQGAP1. Data are representative of four independent experiments. *C*, the amount of pTyr that co-migrates with immunoprecipitated IQGAP1 was quantified with LI-COR Image Studio. The ratio of pTyr to IQGAP1 was graphed, and data were normalized to samples treated with DMSO. Data are means ± S.D. (*error bars*) (*n* = 4). *, *p* < 0.001 by Student's *t* test. *D*, EBC-1 cells were transfected with control (*Ctrl*) siRNA or siRNA against MET. 48 h after transfection, endogenous IQGAP1 was IP. NIRS was used as a control. Samples were analyzed by Western blotting with antibodies to pTyr and IQGAP1 (*left panels*). Equal amounts of protein lysate were processed by Western blotting and incubated with antibodies to IQGAP1, MET, and tubulin (loading control) (*right panel*). Data are representative of three independent experiments. *E*, the graph depicts the ratio of pTyr to immunoprecipitated IQGAP1 as quantified by LI-COR Image Studio. Ratios are normalized to the control siRNA condition. Data are means ± S.D. (*n* = 3). *, *p* < 0.001 by Student's *t* test.

We further validated the role of the MET pathway in a second NSCLC cell line. EBC-1 cells have increased expression of MET and constitutive MET activity (35). Consistent with our observations in H1993 cells, IQGAP1 is constitutively phosphorylated on tyrosine residues in EBC-1 cells (Fig. 5*A*). As was observed in H1993 cells, either crizotinib (Fig. 5 (*B* and *C*) and Fig. S6) or knockdown of MET with siRNA (Fig. 5, *D* and *E*) significantly reduced IQGAP1 tyrosine phosphorylation. Moreover, MS revealed that crizotinib decreased phosphorylation of Tyr-1510 on IQGAP1 by 2.7-fold in EBC-1 cells (Fig. S6). Collectively, these data establish that MET induces tyrosine phosphorylation of IQGAP1 on Tyr-1510 in NSCLC cell lines.

### Mutation of IQGAP1 Tyr-1510 alters AKT activation by HGF

**Figure 6:**
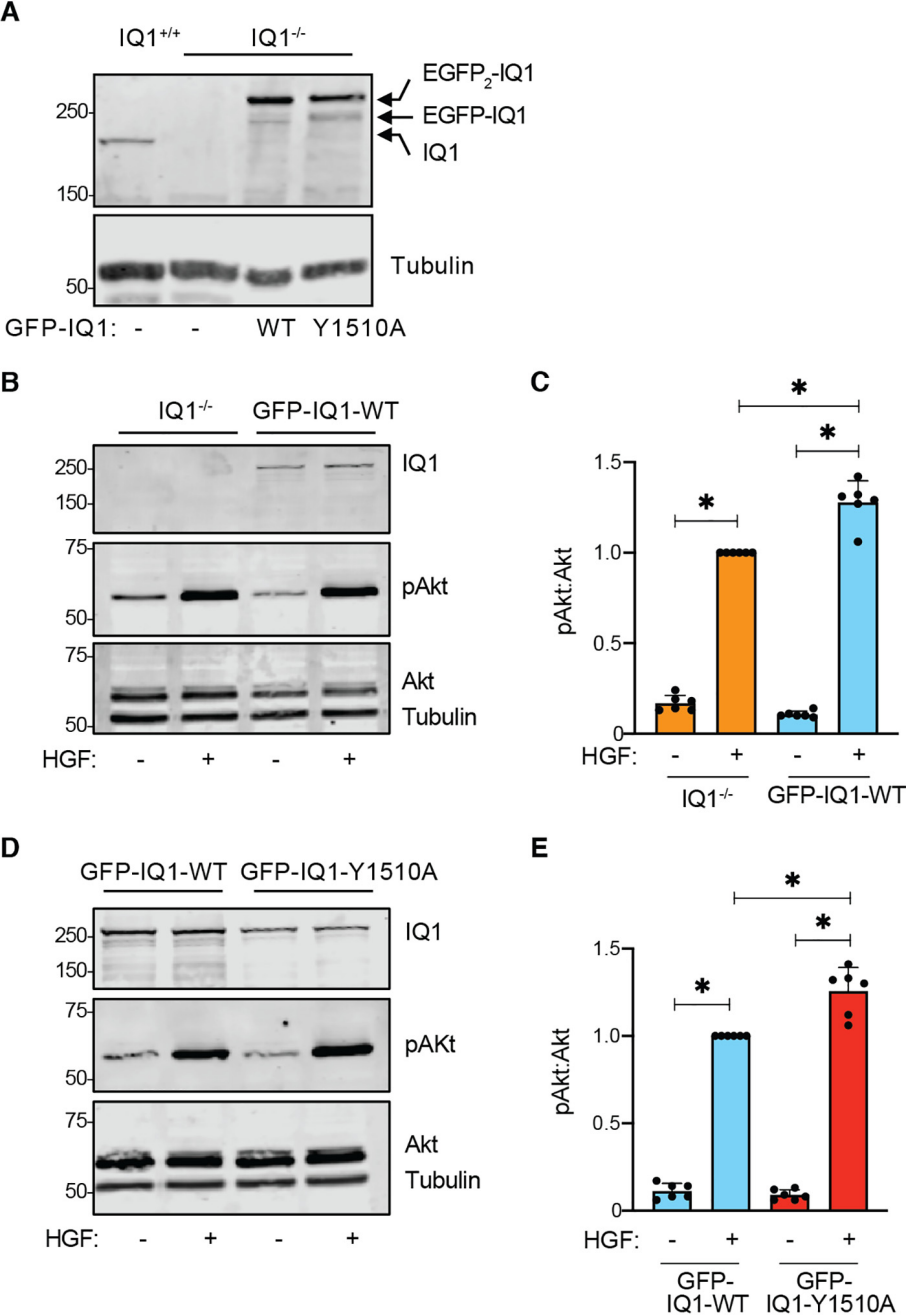
**Mutation of Tyr-1510 of IQGAP1 alters activation of AKT by HGF.***A*, IQGAP1-null MEFs were stably reconstituted with GFP-tagged WT IQGAP1 (*WT*) or IQGAP1 Y1510A (1510A) as described under “Experimental Procedures.” Equal amounts of protein lysate from control MEFs (IQ1^+/+^), IQGAP1-null MEFs (IQ1^−/−^), or IQGAP1-null MEFs reconstituted with one of the GFP-tagged IQGAP1 plasmids were resolved by Western blotting. Blots were probed with antibodies to IQGAP1 (*top*) and tubulin (*bottom*). The label *IQ1* on the *right side* of the blot indicates the position of migration of endogenous IQGAP1. *B*, IQGAP1-null MEFs and IQGAP1-null MEFs reconstituted with GFP-WT-IQGAP1 (*GFP-IQ1-WT*) were serum-starved and incubated with (+) or without (−) 50 ng/ml HGF for 5 min. Equal amounts of protein lysate were analyzed by Western blotting with antibodies to IQGAP1, pAKT, AKT, and tubulin. Data are representative of six independent experiments. *C*, the graph depicts the ratio of pAKT to total AKT as quantified by LI-COR Image Studio. Ratios are normalized to the HGF-treated IQGAP1-null MEF cells. Data are means ± S.D. (*error bars*) (*n* = 6). *, *p* < 0.001 by one-way ANOVA. *D*, IQGAP1-null MEFs stably expressing GFP-tagged WT IQGAP1 (*GFP-IQ1-WT*) or IQGAP1 Y1510A (*GFP-IQ1-Y1510A*) were serum-starved and incubated with (+) or without (−) 50 ng/ml HGF for 5 min. Equal amounts of protein lysate were analyzed by Western blotting. Data are representative of six independent experiments. *E*, the graphs depict the ratio of pAKT to total AKT as quantified by LI-COR Image Studio. Cells reconstituted with WT IQGAP1 and treated with HGF were set as 1. Data are means ± S.D. (*n* = 6). *, *p* < 0.001 by one-way ANOVA.

Published evidence reveals that IQGAP1 is a component of PI3K/AKT signaling (3, 4) and that it is required for maximal activation of AKT by VEGF (13), insulin (3, 27), or EGF (3). Because HGF activates AKT via MET (16, 39), we investigated whether IQGAP1 also participates in this pathway. To do this, we compared the ability of HGF to activate AKT in cells with and without IQGAP1. Mouse embryo fibroblasts (MEFs) derived from IQGAP1-null mice (40) were stably transfected with a plasmid expressing GFP-tagged WT IQGAP1 (Fig. 6*A*). Note that GFP-tagged IQGAP1 routinely exhibits two bands of slightly different molecular mass on SDS-PAGE (Fig. 6*A*), presumably due to cleavage of one GFP molecule from the dual GFP-tagged IQGAP1 constructs (40, 41). We previously documented that the GFP tag does not change the binding of IQGAP1 to several target proteins (10). Parent IQGAP1-null MEFs and IQGAP1-null MEFs reconstituted with GFP-tagged WT IQGAP1 were serum-starved overnight and then incubated with HGF for 5 min. HGF enhances AKT phosphorylation in IQGAP1-null MEFs (Fig. 6, *B* and *C*). When MEFs are reconstituted with IQGAP1, the ability of HGF to activate AKT is significantly greater than that in cells lacking IQGAP1. These data show that IQGAP1 is required for maximal activation of AKT by HGF.

We used this experimental system to investigate the potential biological effect of Tyr-1510 phosphorylation on IQGAP1 in MET signaling. We generated by site-directed mutagenesis a point mutant IQGAP1 construct in which Tyr-1510 was replaced with alanine. The construct, termed IQGAP1 Y1510A, was tagged with GFP. To avoid interference from endogenous IQGAP1, GFP-tagged IQGAP1 Y1510A was stably expressed in IQGAP1-null MEFs. Western blotting revealed that IQGAP1 Y1510A and WT IQGAP1 are expressed at similar levels in the reconstituted IQGAP1-null MEFs (Fig. 6*A*). When cells were incubated with HGF, AKT phosphorylation in MEFs reconstituted with IQGAP1 Y1510A was significantly greater than that in cells reconstituted with WT IQGAP1(Fig. 6, *D* and *E*). Basal AKT phosphorylation did not differ substantially between the two cell lines. These data strongly suggest that phosphorylation of residue Tyr-1510 in IQGAP1 modulates its role in activation of AKT by MET.

### Mutation of IQGAP1 Tyr-1510 enhances HGF-induced cell migration

**Figure 7:**
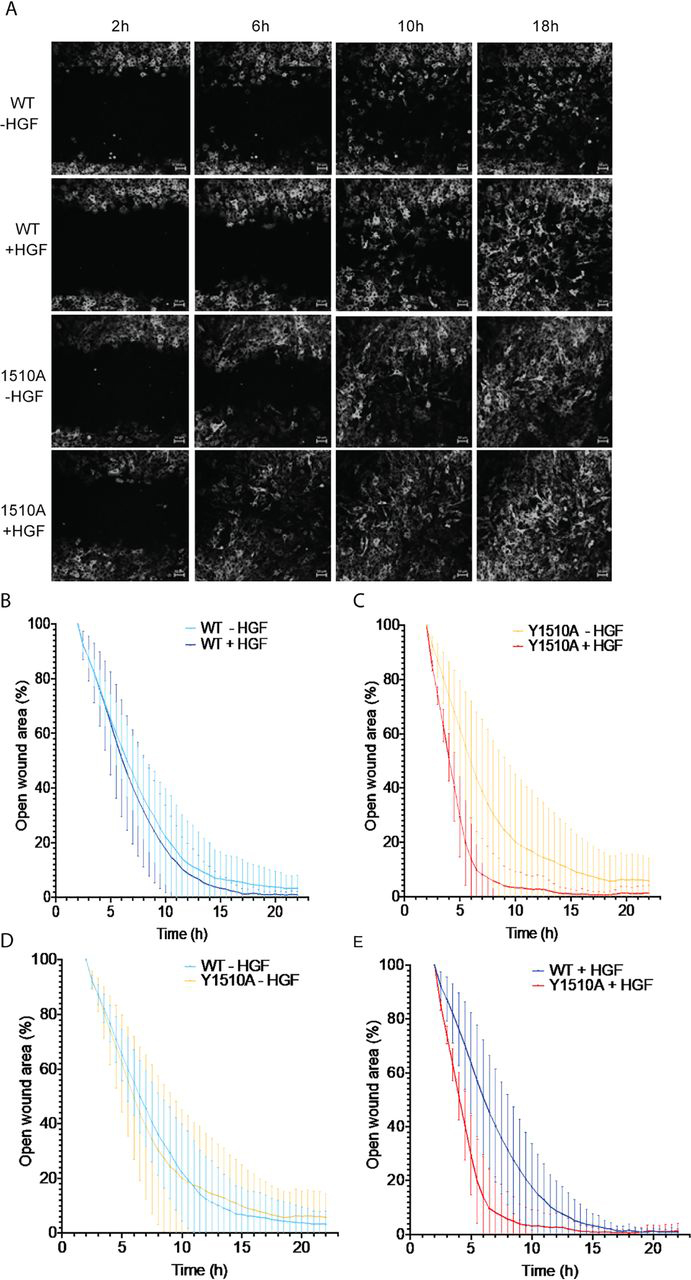
**Mutation of IQGAP1 Tyr-1510 enhances HGF-stimulated cell migration.***A*, expression of GFP-tagged WT IQGAP1 (*WT*) or IQGAP1 Y1510A (*Y1510A*) was induced in IQGAP1-null MEF cells with doxycycline. After serum starvation, cells were incubated with medium containing 0.5% FBS and 100 ng/ml doxycycline, with (+) or without (−) 40 ng/ml HGF, and a wound was generated. Images were taken every 30 min for 24 h. Representative wounds are shown at 2, 6, 10, and 18 h (*scale bar*, 50 μm). *B–E*, open wound areas were analyzed with Fiji/ImageJ as described under “Experimental procedures.” Graphs depict wound closure of MEFs reconstituted with WT IQGAP1 with or without HGF (*B*), IQGAP1 Y1510A with or without HGF (*C*), WT IQGAP1 or IQGAP1 Y1510A without HGF (*D*), or WT IQGAP1 or IQGAP1 Y1510A with HGF (*E*). Means between HGF-stimulated cells with WT IQGAP1 and cells with IQGAP1 Y1510A are significantly different at 2.5 h (*p* < 0.01 by Wilcoxon signed-rank test). Data are expressed as means ± S.D. (*error bars*) (three fields from four independent wells were analyzed; *n* = 12 for all conditions).

MET promotes cell motility (17, 18). To ascertain whether phosphorylation of IQGAP1 has a biological function, we compared the ability of HGF to enhance the migration of cells containing WT IQGAP1 with cells reconstituted with IQGAP1 Y1510A. Cell motility was evaluated by a wound-healing assay using a confluent monolayer of cells. IQGAP1-null MEFs reconstituted with GFP-tagged WT IQGAP1 or IQGAP1 Y1510A were serum-starved overnight and then incubated with or without HGF. The cells along the wound edge were imaged by time-lapse microscopy over a 22-h time period. All of the cells migrated into the wound by cell spreading, not cell division (Fig. 7*A*). HGF significantly increased migration of cells expressing either WT IQGAP1 or IQGAP1 Y1510A (Fig. 7). Analysis of the data revealed that cells reconstituted with IQGAP1 Y1510A reduced the width of the wound more quickly than cells reconstituted with WT IQGAP1 in the presence—but not the absence—of HGF (Fig. 7). Quantification of the cell-free area of the wound revealed that in the presence of HGF cells expressing IQGAP1 Y1510A reduced the wound area by 49 ± 8.8% (mean ± S.D., *n* = 12) 4 h after the wound was generated (Fig. 7*E*). By contrast, cells reconstituted with WT IQGAP1 achieved 24 ± 13.5% (mean ± S.D., *n* = 12 closure) at 4 h. At 6.5 h, cells expressing IQGAP1 Y1510A had 90 ± 14.3% closure, whereas WT IQGAP1 had closed the wound by only 55 ± 21.0% (Fig. 7*E*). These data strongly suggest that phosphorylation of IQGAP1 influences the effects of HGF on cell function.

## Discussion

The pleiotropic effects of IQGAP1 are mediated by its ability to associate with multiple, diverse cellular proteins. IQGAP1 functions as a scaffold protein by assembling components of several of signaling pathways (42), which enables it to participate in essential cellular processes (7, 8). Many factors, such as subcellular location, cell type, and binding affinities, may influence which IQGAP1 complexes are formed (7). In addition, intracellular signaling molecules regulate the association between IQGAP1 and its binding partners. For example, manipulation of intracellular free Ca^2+^ concentrations alters the interaction between IQGAP1 and B-RAF, thereby changing the ability of EGF to activate B-RAF (43). Ca^2+^ also abrogates the binding between IQGAP1 and EGFR in cell lysates (9). Similarly, when calmodulin binds to IQGAP1, the latter has reduced interactions with a number of other proteins, including CDC42 (44, 45) E-cadherin (46), and RAP1 (47). In MCF-7 human breast epithelial cells, estradiol attenuates the interaction between IQGAP1 and estrogen receptor-α, thereby modulating transcriptional function of the receptor (48). Another fundamental mechanism by which protein function is regulated is by post-translational modifications. The human proteome contains hundreds of different types of post-translational modifications, although only a few have been studied extensively (49). In this context, the role of post-translational modifications in regulating IQGAP1 functions has generated interest over the last few years (10, 41, 50).

A few publications have examined how post-translational modifications influence IQGAP1 function. For example, a recent study identified specific residues that are ubiquitinated on IQGAP1, among which are Lys-1155 and Lys-1230 (41). Importantly, replacement of these two lysines with arginine significantly enhanced the association of IQGAP1 with CDC42 and RAC1. Moreover, cells reconstituted with the mutant IQGAP1 protein exhibited both increased active CDC42 and markedly enhanced cell migration (41). By contrast, SUMOylation of IQGAP1 on Lys-1445 is proposed to stabilize the IQGAP1 protein by inhibiting its ubiquitination (51), whereas inhibition of ISGylation of IQGAP1 enhanced CDC42 activity (50). Very few studies have described functions for phosphorylation of IQGAP proteins. When the phosphorylated Ser and Thr sites on the IQGAP-related protein 1 (Iqg1) in *Candida albicans* are mutated, Iqg1 is stabilized, assembly of protein complexes is altered, and cells display abnormal cytokinesis (52). Although multiple phosphorylation sites on IQGAP1 have been identified in phosphoproteomic studies in mammalian cell lines, little is known about the kinases that catalyze these phosphorylations or the functional effects. The best-characterized is the phosphorylation of IQGAP1 on Ser-1441 and Ser-1443, which is promoted by EGF via PKC (9). Phosphorylation of these serine residues altered the ability of IQGAP1 to regulate the cytoskeleton and modulated neurite outgrowth (10). No published study has focused on identifying the tyrosine phosphorylation sites of IQGAP1 and investigating the biological consequences. To address this, we examined tyrosine phosphorylation of IQGAP1.

By inhibiting intracellular dephosphorylation of phosphotyrosine with vanadate, we identified several tyrosine residues that are phosphorylated on IQGAP1 and IQGAP2. Four phosphorylated tyrosine residues were found on IQGAP1 (Tyr-172, Tyr-654, Tyr-855, and Tyr-1510) and three on IQGAP2 (Tyr-14, Tyr-93, and Tyr-770). Most of these tyrosines are in the N-terminal half of the proteins. Phosphoproteomic studies have previously identified all of these phosphorylation sites (11). The only conserved tyrosine that is phosphorylated is Tyr-855 in IQGAP1, which corresponds to Tyr-770 in IQGAP2. All of the tyrosines phosphorylated on IQGAP2 are in the N-terminal half of the protein; two sites are near or in the CHD (Tyr-14 and Tyr-93), whereas the other is in the IQ domain (Tyr-770) (Fig. S3). The tyrosine phosphorylation sites in IQGAP1 are adjacent to the CHD (Tyr-172), between the CHD and WW domain (Tyr-654), in the IQ domain (Tyr-855), and in the RGCT (Tyr-1510) (see Fig. 1*C*). Each of these regions of IQGAP1 mediates distinct interactions: for example, the CHD binds to actin (45, 53, 54); the coiled-coil region between the CHD and WW binds to FOXO (55); the IQ domain is where calmodulin (56), extracellular signal–regulated kinase (1, 2), AKT (4), and PI3K (3) bind; and the RGCT interacts with Dia1 (57), IRS-1 (27), and CLIP-170 (58).

Interestingly, the most commonly identified phosphorylation site on IQGAP1 is Tyr-1510, which has been detected in 937 phosphoproteomic analyses (11). Tyr-1510 of IQGAP1 is conserved in mammals and in many reptiles and birds. Nevertheless, Tyr-1510 of IQGAP1 does not align with a tyrosine residue in IQGAP2 or IQGAP3. Under our experimental conditions, we observed phosphorylation of Tyr-1510 using several independent but complementary techniques. Tyr-1510 was one of the residues phosphorylated on IQGAP1 that we identified by MS in MDA-MB-231 cells with inhibition of phosphatase by vanadate.

Importantly, we identified two kinases that induce Tyr-1510 phosphorylation, namely MET and c-Src. Transfection of HEK293 cells with MET or c-Src promoted phosphorylation of IQGAP1 only on Tyr-1510. Similarly, we documented in two malignant lung carcinoma cell lines with enhanced MET activation that IQGAP1 is constitutively phosphorylated exclusively on Tyr-1510. Importantly, we were able to attenuate phosphorylation of Tyr-1510 in IQGAP1 in these NSCLC cells by chemical inhibition of MET or by siRNA-mediated knockdown of MET expression. Altogether, these studies reveal that MET signaling promotes phosphorylation of IQGAP1 exclusively on Tyr-1510. It is intriguing that only a single amino acid residue was phosphorylated with MET or c-Src overexpression. The reason for the phosphorylation of only one of the 62 tyrosine residues in IQGAP1 is unknown, but it is consistent with our prior observation that EGF induces phosphorylation of IQGAP1 on only one (or possibly two) amino acids (9).

Global screens of tyrosine-phosphorylated peptides identified Tyr-1510 phosphorylation in fibroblasts overexpressing c-Src (59, 60). Moreover, a prior publication (14) demonstrated that c-Src promotes IQGAP1 tyrosine phosphorylation, but the authors did not determine which residue(s) were phosphorylated. By contrast, few publications have evaluated the participation of IQGAP1 in MET signaling. Initially identified in a screen for oncogenes, MET is overexpressed in numerous cancers (reviewed in Ref. 16), and MET signaling promotes cell proliferation, migration, and metastasis. Two studies performed in human endothelial cells demonstrated that HGF enhances the association of IQGAP1 with several proteins, including cortactin, EB1, and the ASEF1, which promotes endothelial barrier functions. These associations are attenuated by treatment with MET inhibitors (21, 22). Although not examined in these publications, it is possible that IQGAP1 Tyr-1510 phosphorylation may modulate these interactions, as cortactin, EB1, and ASEF1 all require the C-terminal region of IQGAP1 (amino acids 1502–1657) for binding (21, 22).

Downstream of MET, signaling pathways, including the PI3K/AKT cascade, are activated (39). Upon MET activation, PI3K binds directly, or indirectly through adaptor proteins, to the intracellular domain of MET. Next, PI3K produces phosphatidylinositol 3,4,5-trisphosphate that activates AKT. IQGAP1 binds to both PI3K and AKT and is required for maximal AKT activation via receptor tyrosine kinases (3, 13, 27). Given this background, we examined the potential roles of IQGAP1 and IQGAP1 Tyr-1510 phosphorylation in HGF stimulation of AKT. We observed that, analogous to VEGF (13), EGF (3), and insulin (27), IQGAP1 is necessary for HGF to maximally activate AKT. Note that IQGAP1 is not required for activation of AKT by HGF, which is similar to the observations for the other growth factors. To evaluate the role of Tyr-1510 phosphorylation of IQGAP1, we replaced Tyr-1510 with alanine to generate a construct that cannot be phosphorylated on this residue. When IQGAP1-null MEFs were reconstituted with IQGAP1 Y1510A, the ability of HGF to activate AKT was greater than that in cells reconstituted with WT IQGAP1. While the effect was small (phosphorylated AKT was 26% higher), the increase was statistically significant. Taken together, our data reveal that IQGAP1 is required for MET to maximally activate AKT and that phosphorylation of Tyr-1510 of IQGAP1 modulates this effect.

There are several potential mechanisms by which phosphorylation of IQGAP1 on Tyr-1510 could alter PI3K/AKT signaling. IQGAP1 binds directly to both AKT (4) and PI3K (3), the kinase that induces activation of AKT. Incorporation of a phosphate residue on Tyr-1510 may induce interactions of IQGAP1 with other proteins, thereby influencing PI3K and/or AKT interactions. For example, IQGAP1 binds to several proteins, including c-Src (14), ShcA (28), IRS-1 (27), and PI3K (3), which contain SH2 (Src homology 2) or PTB (phosphotyrosine-binding) domains that bind to phosphotyrosine residues (61). All of these proteins influence the PI3K/AKT cascade. Alternatively, phosphorylation may change the tertiary conformation of IQGAP1 and produce an allosteric effect, as has been proposed previously for some other regulators that influence binding of IQGAP1 to selected proteins (4, 45, 54). A third possibility is that phosphorylation may alter IQGAP1 localization. Stimulation with growth factors, including HGF (22), promotes IQGAP1 localization to the plasma membrane (14, 15, 62), whereas deletion of the C-tail of IQGAP1 (which contains Tyr-1510) inhibits this localization (63, 64).

We wanted to ascertain whether phosphorylation of Tyr-1510 on IQGAP1 has a biological function. As mentioned earlier, HGF/MET elicits effects on cells, including the promotion of cell motility (17, 18). Importantly, HGF stimulates cell migration, at least in part, by activation of PI3K/AKT signaling (65, 66, 67). Moreover, our data indicate that IQGAP1 is required for MET to maximally activate AKT and that phosphorylation of Tyr-1510 of IQGAP1 modulates this effect. Therefore, we assessed cell motility as the biological readout of IQGAP1 tyrosine phosphorylation. As anticipated, HGF enhanced motility of IQGAP1-null MEFs reconstituted with either WT IQGAP1 or IQGAP1 Y1510A. Importantly, HGF reduced the width of the wound more quickly with cells expressing IQGAP1 Y1510A than cells with WT IQGAP1. These data, which are consistent with the enhanced ability of HGF to activate AKT in cells containing IQGAP1 Y1510A, reveal that Tyr-1510 of IQGAP1 has a biological function. Note that tyrosine phosphorylation of IQGAP1 is partially, but not completely, responsible for the migratory response to HGF.

In summary, our study shows that Tyr-1510 phosphorylation of IQGAP1 is under the regulation of MET signaling. We observed that IQGAP1 is constitutively phosphorylated on Tyr-1510 in NSCLC cells that overexpress MET. MS analyses of protein phosphorylation in mouse models of human lung cancers, including those with enhanced EGFR (68) and MET (69) signaling, have identified phosphorylation of Tyr-1510 on IQGAP1. Taken together with our data, these observations suggest that IQGAP1 Tyr-1510 phosphorylation is likely to occur in some lung carcinomas in which MET is up-regulated. Many patients with lung tumors that are resistant to EGFR inhibitors have increased MET (70, 71, 72). Phosphorylation of IQGAP1 on Tyr-1510 in some tumors may alter AKT (and perhaps other signaling pathways) to influence tumor growth or progression. The data presented in our study add to the burgeoning evidence that IQGAP1 may be a potential therapeutic target in selected malignancies.

## Experimental procedures

### Antibodies and reagents

Antibodies and dilutions used are listed in Table 1. Nonimmune rabbit serum (catalog no. 10510) was purchased from Thermo Fisher Scientific. Blocking buffer and secondary antibodies were purchased from LI-COR Biosciences. Purified recombinant MET (catalog no. 14-526) and c-Src (catalog no. 14-326) were from Sigma–Aldrich. Both kinases were His-tagged, recombinant proteins expressed by baculovirus in Sf21 insect cells and purified using Ni^2+^/nitrilotriacetic acid-agarose. BSA was from New England Biolabs (catalog no. B9000S). Cell culture reagents Dulbecco's modified Eagle's medium (DMEM) (catalog no. 11965-092), Roswell Park Memorial Institute 1640 (RPMI 1640) (catalog no. 11875-093), fetal bovine serum (FBS) (catalog no. 16140), and OPTI-MEM (catalog no. 31987-070) were purchased from Gibco. Lipofectamine 2000 (catalog no. 11668019), Lipofectamine RNAiMAX (catalog no. 13778150), Lipofectamine LTX (catalogue no. 11668019), hygromycin (catalog no. 10687010), doxycycline (catalog no. BP26531), and DMSO (catalog no. BP231-100) were from Thermo Fisher Scientific. Crizotinib (catalog no. S1068), afatanib (catalog no. S7810), and PP2 (catalog no. S7008) were obtained from Selleck Chemicals. Sodium orthovanadate (catalog no. S6508-10G) was from Sigma. Recombinant human HGF (catalog no. 294HG005CF) was purchased from R&D Systems. The colloidal blue staining kit was purchased from Invitrogen (catalog no. LC6025). GSH-Sepharose and protein A-Sepharose beads were purchased from GE Healthcare. Protein concentrations were measured using Bio-Rad protein assay dye reagent concentrate (catalog no. 500-0006).

**Table 1:** Antibodies used in this study

Protein detected	Source/reference	Dilution
AKT	Cell Signaling Technology, 2920S	1:1000
pAKT Ser-473	Cell Signaling Technology, 4060S	1:1000
β-Tubulin	Sigma–Aldrich, T5201-200UL	1:5000
c-MET	Cell Signaling Technology, 8198S	1:1000
c-Src	Cell Signaling Technology, 2108S	1:1000
GFP	Cell Signaling Technology, 2955S	1:1000
IQGAP1 for WB^a^	Millipore, 05-504	1:1000
IQGAP1 for WB and immunoprecipitation, IQGAP2 for WB	Antiserum (45), Santa Cruz, sc-55525	1:1000
IQGAP2 for immunoprecipitation	Antiserum (78)	1:1000
Myc	Millipore, 06-549	1:1000
Phosphotyrosine	Cell Signaling Technology, 8954S	1:2000


a WB, Western blotting.

### Plasmid construction and expression

The construction of Myc-tagged and GST-tagged IQGAP1 has been described previously (40, 45). Myc-HER2 (34), Myc-IR_cyt_ (the cytoplasmic domain of the human insulin receptor, comprising amino acids 980–1382) (27), and GFP-Axl (33) were described previously. Plasmids for expression of MET (pT3-EF1aH c-Met was a gift from Xin Chen, plasmid no. 86498 (73), and c-Src (pcDNA3 c-SRC (WT) was a gift from Robert Lefkowitz, plasmid no. 42202 (74)) were obtained from Addgene. The GFP-tagged cytoplasmic domain of MET (amino acids 956–1390) plasmid was made by amplifying MET from the pT3-EFa1H c-MET plasmid using the following primers: forward: 5′-CGGGATCCAAAAAGAGAAAGCAAATTAA-AGATCTGGGC-3′ and reverse 5′-CGGAATTCAAGCTTCTATGATGTCTCCCAGAACGAGGCTG-3′. Then PCR pro-ducts were cut with BamHI and EcoRI and inserted into pEGFP-C1 (Clontech) at BglII and EcoRI sites. Site-directed mutagenesis was performed on IQGAP1 to replace tyrosine by alanine on residue 1510 using the following primers: forward, 5′-GAAACTGCAACAGACAGCCGCTGCTCTGAAGTC-3′; reverse, 5′-GAG-TTCAGAGCAGCGGCTGTCTGTTGCAGTTTC-3′. Mutagenesis was performed on pBlueScript vectors containing the IQGAP1 ClaI/XbaI region (amino acids 1193–1657), essentially as described previously (41, 56). The pBlueScript vectors was digested with ClaI and XbaI, and the resultant fragments were inserted into the pcDNA3-Myc-IQGAP1 plasmid at ClaI/XbaI sites to generate Myc-IQGAP1 Y1510A construct. To make the GFP-IQGAP1 Y1510A construct, the plasmid pEGFPx2-IQGAP1 (40) was cut with NheI. The fragment generated, containing the EGFPx2-IQGAP1-N se-quence, was inserted into vector pEN-TmiRc3 at the SpeI site. Then the plasmid pCDNA3-myc-IQGAP1 Y1510A was cut with PacI/XbaI. The resultant fragment, containing the EGFPx2-IQGAP1-C sequence, was inserted into pEN-EGFPx2-IQGAP1-N at PacI/XbaI sites to generate full-length pEN-EGFPx2-IQGAP1 Y1510A. Recombination of pEN-EGFPx2-IQGAP1 Y1510A and pSLIK-hygro (gift from I. D. Fraser (75)) was accomplished using the Gateway IR Clonase II enzyme mix kit (Invitrogen). The pcDNA3-Myc IQGAP1 Y1510A vector was digested, and the fragment containing the Y1510A mutation was inserted into the pSLIK-hygro-EGFPx2-IQGAP1 vector essentially as described (41). All sequences were confirmed by DNA sequencing, and all of the proteins migrated to the expected position on SDS-PAGE.

### Cell culture and treatments

MDA-MB-231, HEK293, and NCI-H1993 cells were obtained from American Type Culture Collection. EBC-1 cells were obtained from the Japanese Collection of Research Bioresources Cell Bank. Immortalized MEF cells isolated from embryos of IQGAP1-null and control mice were described previously (43). MDA-MB-231, HEK293, and MEF cells were cultured in DMEM plus 10% FBS, whereas H1993 and EBC-1 cells were cultured in RPMI 1640 containing 10% FBS. Where indicated, Na_3_VO_4_ (final concentration 2 mm) was added to the cell culture plates for 16 h. For inhibitor treatment, crizotinib (100 nm), afatanib (5 μm), or PP2 (10 μm) (all inhibitors were dissolved in DMSO, with final concentrations indicated in the figure legends) or DMSO alone (vehicle control) was added to the cells 24 h before immunoprecipitation. For siRNA transfection, 10 µl of 10 mm control (Silencer Negative Control, Thermo Fisher Scientific, catalog no. AM4635) or c-MET (Santa Cruz Biotechnology, catalog no. sc-29397) siRNA complexes were prepared in Opti-MEM with 20 µl of Lipofectamine RNAiMAX for reverse transfection with H1993 and EBC-1 cells following the manufacturer's protocol. Immunoprecipitation was performed 48 h after siRNA transfection. For transient transfections, HEK293 cells were plated in 10-cm dishes in Opti-MEM, and 4 µg of DNA was transfected using Lipofectamine 2000 following the manufacturer's protocol. Cells were harvested for immunoprecipitation 48 h after transfection.

### Immunoprecipitation for detection of tyrosine phosphorylation

Cells were lysed in lysis buffer (50 mm Tris-HCl, pH 7.4, 150 mm NaCl, 1% Triton X-100) containing HALT protease and phosphatase inhibitors (Thermo Scientific, catalog no. 78444). Lysates were subjected to sonication for 10 s, and insoluble material was precipitated by centrifugation at 20,000 × *g* for 10 min at 4 °C. Supernatants were precleared with GSH-Sepharose beads for 1 h at 4 °C. 10 µl of anti-IQGAP1 antiserum, anti-IQGAP2 antiserum, or NIRS was bound to protein A–Sepharose beads for 1 h. Protein concentrations of cell lysates were measured with Bio-Rad protein assay dye reagent kit, and equal amounts of protein lysate were used for immunoprecipitation. Precleared lysates were then incubated with antibodies bound to protein A beads for 3 h at 4 °C. After washing the beads five times with lysis buffer, samples were resolved by SDS-PAGE and transferred to a PVDF membrane. The membrane was incubated with blocking buffer (LI-COR Biosciences) for 1 h at 22 °C, and blots were probed with the primary antibodies indicated in the figure legends. The membrane was incubated with IRDye-conjugated secondary antibodies for 1 h, and antigen-antibody complexes were detected using the Odyssey imaging system (LI-COR Biosciences).

### In vitro protein phosphorylation assay

GST-tagged IQGAP1 was purified from *E. coli* essentially as described previously (45). 400 ng of purified GST-IQGAP1 was incubated in 50 mm HEPES, pH 7.5, 15 mm MgCl_2_, 1 mm EGTA, 10% glycerol, 10 mm DTT, 0.1 mm ATP. The reaction was initiated by adding 1 µg of pure, recombinant MET, c-Src, or BSA. After 20 min at 30 °C, the reaction was terminated by the addition of boiling SDS loading buffer (76). Samples were resolved by 10% SDS-PAGE. The gel was cut at ∼100 kDa. The top portion was transferred to PVDF membranes, and the blots were probed with antibodies to IQGAP1 or phosphotyrosine. The lower portion of the gel was stained with Coomassie Blue to visualize the kinases.

### MS

Endogenous IQGAP1, immunopurified from treated or control MDA-MB-231, H1993, EBC-1, and HEK293 cells, was separated by SDS-PAGE, and the gels were stained with Colloidal Coomassie. For each treatment condition (orthovanadate, crizotinib, siRNA, overexpression), a gel band corresponding to the region of migration of IQGAP1 (∼190 kDa) was excised from the stained gel, reduced with 5 mm tris(2-carboxyethyl)phosphine, alkylated with 10 mm chloroacetamide, and digested overnight with 100 ng of trypsin at 37 °C. The gel bands were extracted with 50% acetonitrile, 0.2% formic acid, and the resultant peptide samples were dried, resuspended in 0.1% formic acid and 0.05% TFA, and injected for LC–MS/MS analysis. For initial mapping of phosphotyrosine sites following orthovandate treatment in MDA-MB-231 cells, LC-MS/MS analysis was performed on a LTQ-Orbitrap Velos Pro (Thermo Scientific) mass spectrometer equipped with an Agilent 1100 Nano HPLC system and a nanospray source. Peptides containing 0.1% formic acid and 0.05% TFA were loaded onto a 200 μm × 5-mm trap column with 0.05% heptafluorobutyric acid (HFBA) at 10 µl/min. After 5 min, the trap is put in line with a 100 μm × 5-cm analytical column using mobile phases A (0.2% formic acid in water) and B (0.2% formic acid in acetonitrile) at a flow rate of 0.5 µl/min. Both trap and analytical columns are monolithic polystyrene-divinylbenzene (PS-DVB) polymer type columns (Dionex). Tryptic peptides were separated using a 110-min gradient of 2–30% acetonitrile, 0.2% formic acid. An additional 30 min, which includes sample loading (5 min), end ramp from 30% B to 95% B (5 min), end hold at 95% B (5 min), and column re-equilibration at 2% B (15 min), is added to each gradient program to give the total run time. The analytical column is interfaced via high-voltage liquid contact made through an SS union to a 50-μm fused silica emitter (New Objective) pulled to 15 μm at the tip. MS data were acquired from *m*/*z* 400 to 2000 at a resolution of 30,000. Data-dependent MS/MS acquisition was set to trigger up to 10 ion trap collision-induced dissociation spectra per MS scan using a precursor isolation window of 2.0 *m*/*z* and normalized collision energy of 35. The MS/MS AGC target value was set at 1e4 with a maximum injection time of 100 ms. Dynamic exclusion was set for 30 s. These uninterpreted tandem MS spectra were processed using Mascot Distiller (Matrix Science version 2.7.1.0) and searched for peptide matches against the UniProt_Human (version 2014_03) protein sequence database using Mascot (Matrix Science, version 2.6.0). 65,630 total protein sequences were searched using trypsin enzyme specificity with a possibility of two missed cleavages and a precursor mass tolerance of ±5 ppm and a fragment mass tolerance of 0.8 Da. Carbamidomethylation was selected as a fixed modification on Cys residues. Oxidation (M), Acetyl (Protein N-term), Phospho (ST), and Phospho (Y) were selected as variable modifications. Phosphopeptides assigned with individual MASCOT ion scores >26 (*p* < 0.05) were considered, and these MS/MS spectra were further reviewed manually to confirm tyrosine phosphorylation site identification (Figs. S2 and S5).

For mapping of IQGAP1 phosphotyrosine sites following pharmacologic and genetic manipulation in H1993, EBC-1, and HEK293 cells, the resultant peptide samples were injected on an Easy-nLC 1000 UHPLC system (Thermo Scientific) interfaced to a Q-Exactive Hybrid Quadrupole-Orbitrap mass spectrometer (Thermo Scientific). Tryptic peptides were separated on a 25 cm × 75-μm inner diameter, PepMap C18, 2-μm particle column (Thermo Scientific) using a 40-min gradient of 2–30% acetonitrile, 0.2% formic acid and a flow of 300 nl/min. MS-based peptide sequencing data were acquired by MS using a top 10 data-dependent LC-MS/MS method. Full MS data were collected in profile mode from 400–1800 *m*/*z* at a resolution of 70,000 using an AGC target value of 1e6 with a maximum injection time of 200 ms. The top 10 peptide ions were then isolated using a 1.2 *m*/*z* window and fragmented with a normalized collision energy of 30. MS/MS data were collected using an AGC target value of 5e4 with a maximum injection time of 200 ms and a fixed first mass at 145 Da. A dynamic exclusion of 30 s was applied. These uninterpreted tandem MS spectra were processed using Mascot Distiller (Matrix Science, version 2.7.1.0) and searched for peptide matches against the UniProt_Human (version 2014_03) protein sequence database using Mascot (Matrix Science, version 2.6.0). 65,630 total protein sequences were searched using trypsin enzyme specificity with a possibility of two missed cleavages, a precursor mass tolerance of ±5 ppm, and a fragment mass tolerance of 0.02 Da. Carbamidomethylation was selected as fixed modification on Cys residues. Oxidation (M), Acetyl (Protein N-term), Phospho (ST), and Phospho (Y) were selected as variable modifications. Phosphopeptides assigned with individual MASCOT ions scores >25 (*p* < 0.05) were considered, and these MS/MS spectra were further reviewed manually to confirm tyrosine phosphorylation site identification.

### Generation of IQGAP1 knockout MEF cells with stable expression of IQGAP1

IQGAP1-null MEF cells with stable expression of WT 2xEGFP-IQGAP1 were described previously (41). For stable expression of 2xEGFP-IQGAP1 Y1510A, IQGAP1-null MEFs were transfected with the pSLIK-hygro-EGFPx2-IQGAP1 Y1510A plasmid using Lipofectamine LTX following the manufacturer's instructions. After 48 h, cells were selected using 200 µg/ml hygromycin. Cells were cultured in 200 µl/ml hygromycin and 1 µl/ml doxycycline to maintain expression of GFP-tagged IQGAP1. Expression of GFP-IQGAP1 was confirmed by Western blotting.

### HGF-stimulated AKT signaling

MEF cells were grown in DMEM containing 10% FBS. Expression of WT IQGAP1 or IQGAP1 Y1510A in IQGAP1-null MEFs was induced by adding 1 µg/ml doxycycline to DMEM containing 10% FBS and 200 µl/ml hygromycin. Cells were plated in 6-well dishes at 80% confluence. The following day, cells were washed with PBS and starved overnight in serum-free DMEM. Cells were treated with or without 50 ng/ml HGF for 5 min, washed with PBS, and collected in lysis buffer. After sonication, insoluble material was precipitated by centrifugation at 20,000 × *g* for 10 min at 4 °C. Protein concentrations of cell lysates were measured with a Bio-Rad protein assay dye reagent kit, and equal amounts of protein lysate were resolved by SDS-PAGE and Western blotting. Membranes were probed with antibodies to phospho-AKT, total AKT, IQGAP1, and tubulin.

### Migration assay

Cell migration was evaluated essentially as described previously (41). WT IQGAP1 or IQGAP1 Y1510A expression was induced in IQGAP1-null MEF cells by adding 1 μg/ml doxycycline to DMEM containing 10% FBS. Cells were suspended to a concentration of 5.0 × 10^5^ cells/ml, and 70 μl of cell suspension (35,000 cells) was added to each chamber of an Ibidi Culture-Insert (Ibidi, catalog no. 81176). Cells were allowed to attach to the dish and to reach confluence. After serum starvation for 12 h, the silicone inserts were removed with sterile forceps, and the medium was replaced with DMEM containing 0.5% FBS, 100 ng/ml doxycycline, and 40 ng/ml HGF. Confocal images were acquired every 30 min for 22 h using a Zeiss LSM780 microscope equipped with a ×20 plan-apochromat (numerical aperture 0.8) objective lens, transmitted light detector (T-PMT), and OKO Laboratory Bold Line stage top incubator to control temperature at 37 °C, CO_2_ at 5%, and humidity. Differential interference contrast images were collected simultaneously with confocal images of E fluorescence using 0.461-μm *x*-*y* pixel size, 5.0-μm optical section thickness, and 12-bit data depth. Image analysis was performed using FIJI/ImageJ (77). Brightfield images were processed using a 20 pixel kernel Gaussian blur pseudo-flatfield correction, followed by a 10 pixel kernel gradient morphological filter. The resultant edges of the cell monolayer were then segmented by intensity and filtering for particles >1000 μm^2^. The empty area between the two edges was quantified at each time point.

### Statistical analysis

All statistical analysis was performed using Prism7 (GraphPad) software. Pertinent bands on Western blots were quantified with Image Studio 2.0 (LI-COR Biosciences) according to the manufacturer's instructions. The amount of tyrosine-phosphorylated protein was corrected for the amount of IQGAP1 in the same sample. The amount of pAKT was corrected for the amount of AKT in the same sample.

## Data availability

MS data have been deposited in the MassIVE database under accession numbers MSV000086088 (vanadate) and MSV000086089 (MET pathway).
