# Supplementary material for: Tyrosine phosphorylation of the scaffold protein IQGAP1 in the MET pathway alters function
Source: J Biol Chem. 2025 Jan 13;295(52):18105–21. doi: 10.1074/jbc.RA120.015891 (PMC11843583; doi:10.1074/jbc.RA120.015891)
Supplement: Supplementary file 1 [file mmc1.docx]

**Supporting information**

**
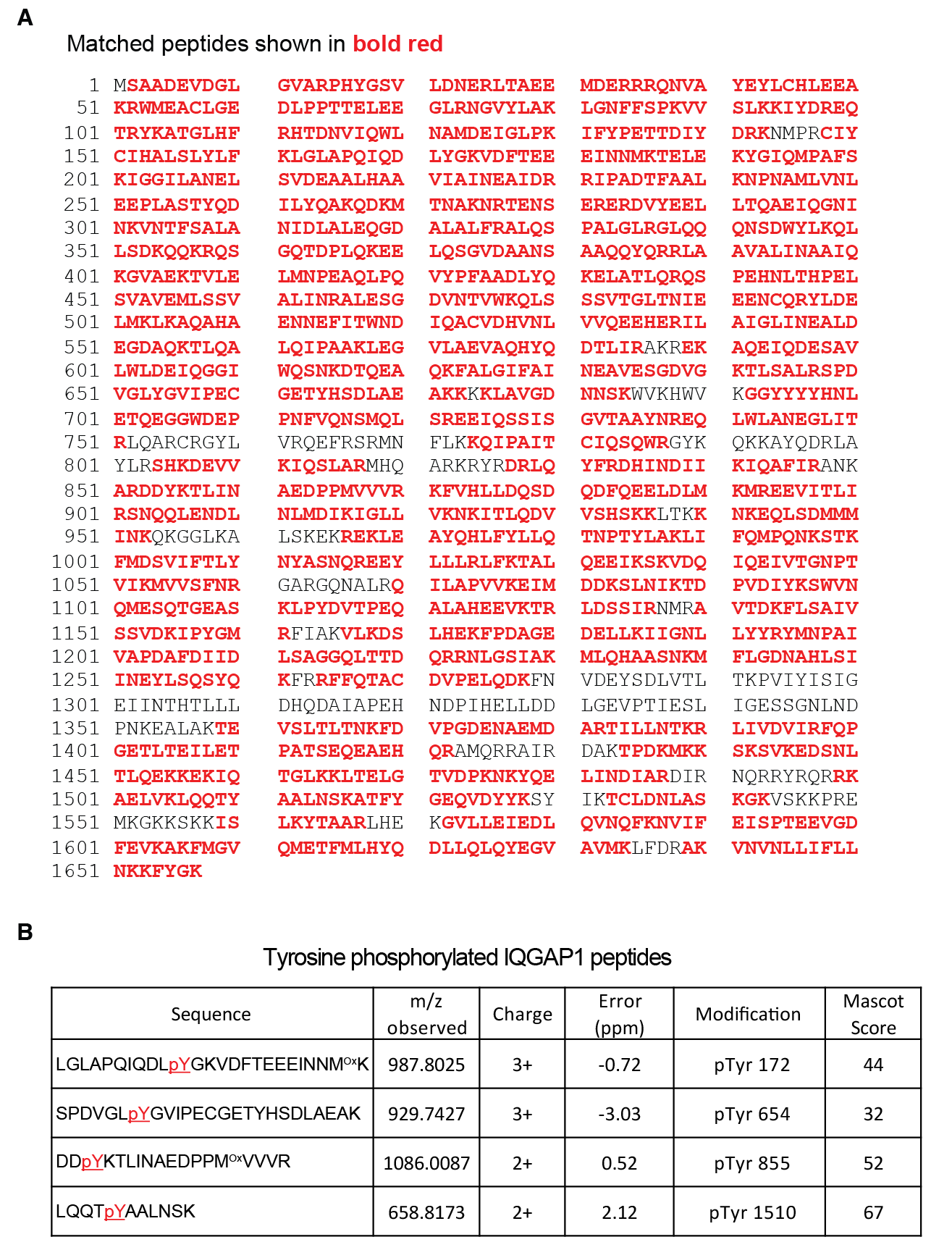
**

**Figure S1. Summary of IQGAP1 phosphorylation in MDA-MB-231 cells treated with vanadate.**

**(**A) Sequence coverage for IQGAP1 immunoprecipitated from MDA-MB-231 cells treated with Na3VO4. This is typical of sequence coverage obtained throughout this work. Sequence coverage includes 53/62 tyrosine residues (85%). (B) Table of tyrosine phosphorylated peptides on IQGAP1 identified from vanadate treated MDA-MB-231 cells.

**
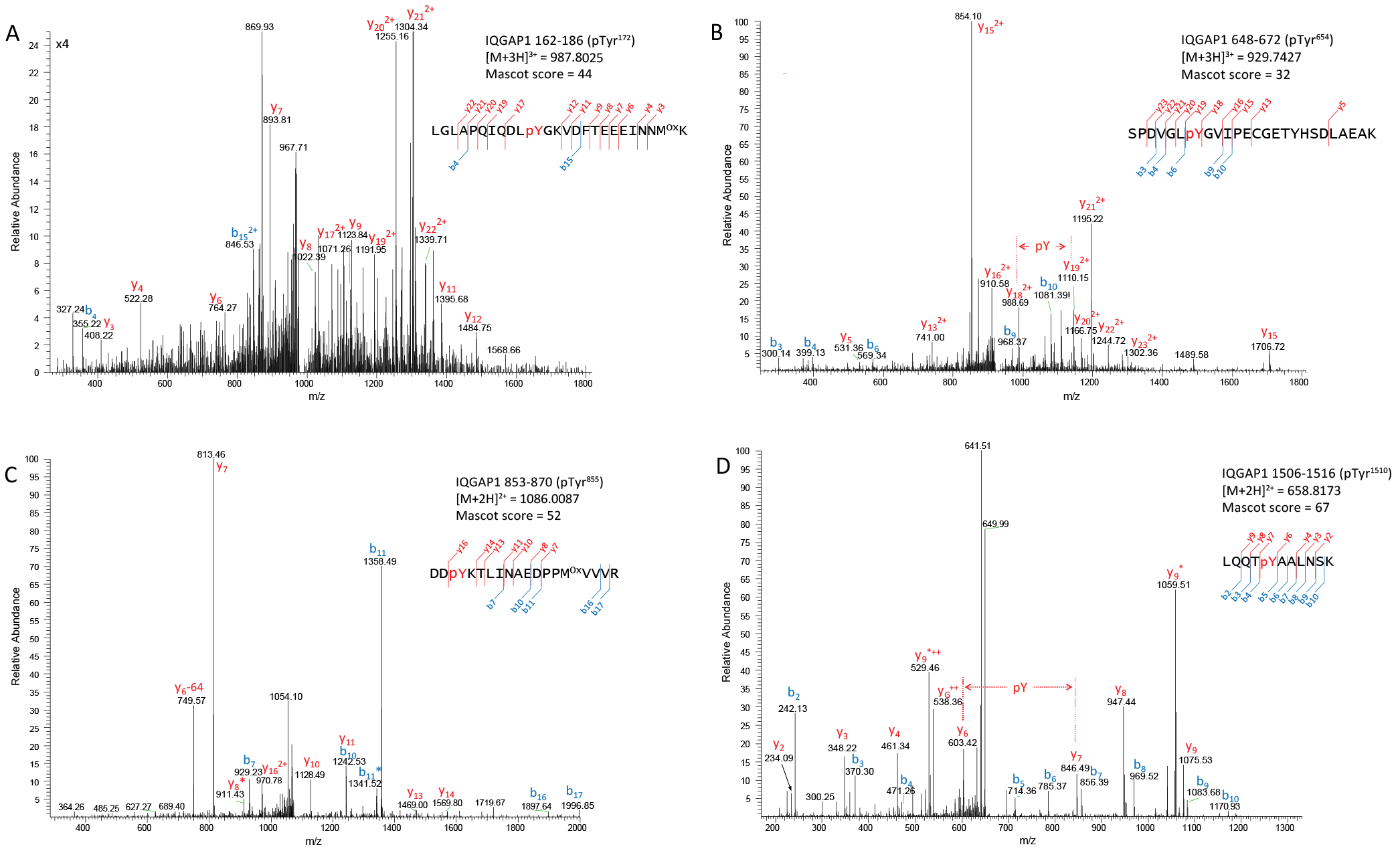
**

**Figure S2. Mass spectrometric spectra for phosphorylated IQGAP1 peptides in MDA-MB-231.**

LC-MS/MS analysis of the in-gel tryptic digested samples allowed the identification of tyrosine phosphorylated residues from MDA-MB-231 cells treated with vanadate. A representative MS/MS spectrum for each of the tyrosine phosphorylated peptides is shown for the following regions of IQGAP1: (A) amino acids 162-186 containing phosphorylated Tyr-172, (B) amino acids 648-672 containing phosphorylated Tyr-654, (C) amino acids 853-870 containing phosphorylated Tyr-855, (D) amino acids 1506-1516 containing phosphorylated Tyr-1510.

**
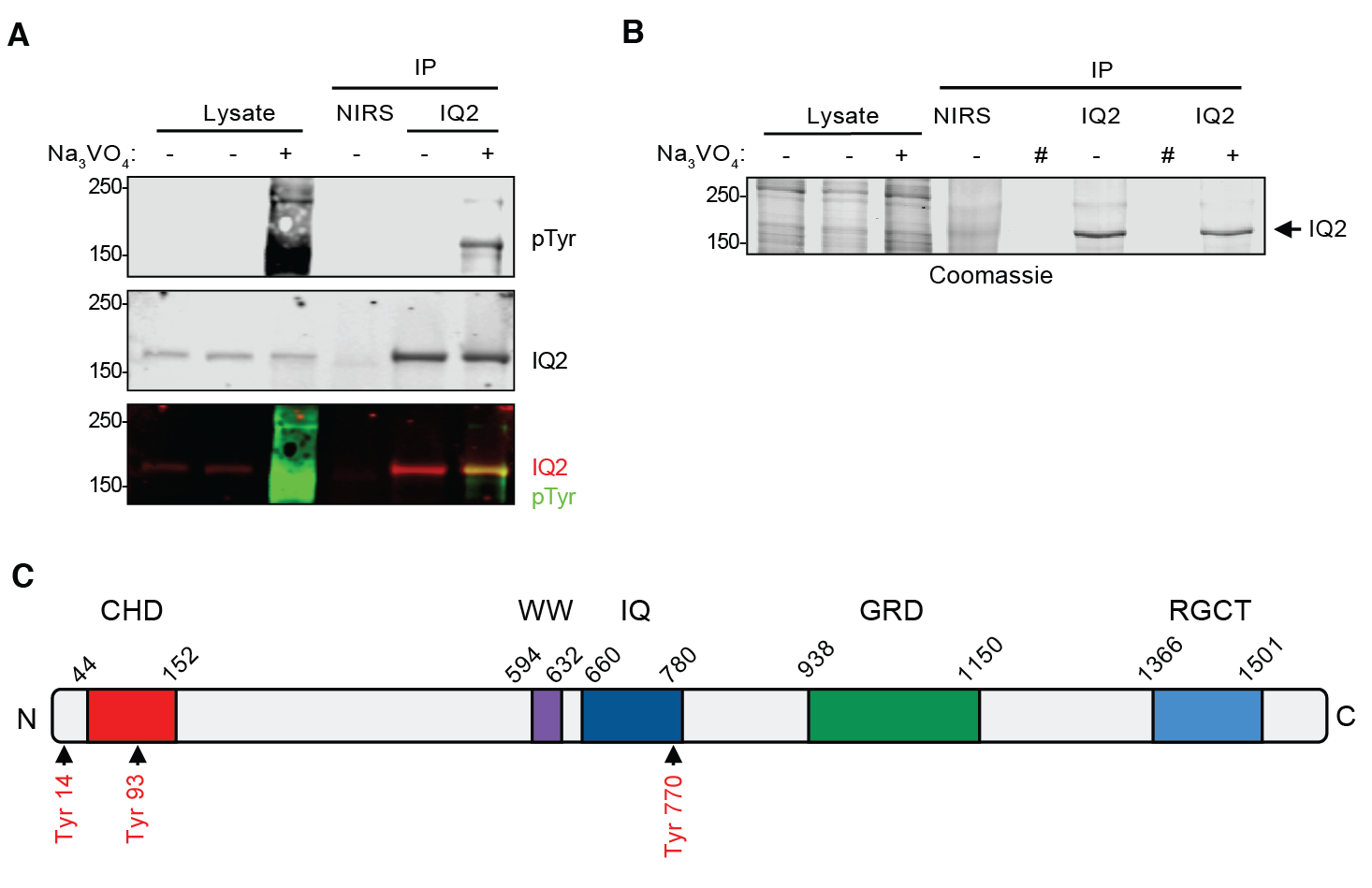
**

**Figure S3. IQGAP2 is tyrosine phosphorylated in HepG2 cells treated with vanadate.**

(A) HepG2 cells were treated with (+) or without (-) 2 mM Na_3_VO_4_ for 16 h. Endogenous IQGAP2 was immunoprecipitated (IP) with anti-IQGAP2 (IQ2) antibody from cell lysates. Non-immune rabbit serum (NIRS) was used as a control. Lysate and IP samples were processed by Western blotting and probed with antibodies to phospho-tyrosine (pTyr, top panel) and IQGAP2 (IQ2, middle panel). The overlap between IQGAP2 (red) and pTyr (green) signals is visible in the merged image (bottom panel). Data are representative of 3 independent experiments. (B) HepG2 cells were treated as in panel A. IQGAP2 was immunoprecipitated with anti-IQGAP2 antibodies and samples were processed as in panel A. Empty lanes are designated by #. Samples were resolved by SDS-PAGE and the gel was stained with Colloidal Coomassie. IQGAP2 bands (arrow) were excised from the gel and analyzed by mass spectrometry. (C) Schematic of tyrosine phosphorylation sites on IQGAP2 identified by mass spectrometry. IQGAP2 contains 5 domains: a calponin-homology domain (CHD), a tryptophan-containing domain (WW), an IQ domain (IQ), a GAP-related domain (GRD) and a RasGAP_C-terminus (RGCT). The phosphorylated tyrosine residues (red) are labelled.


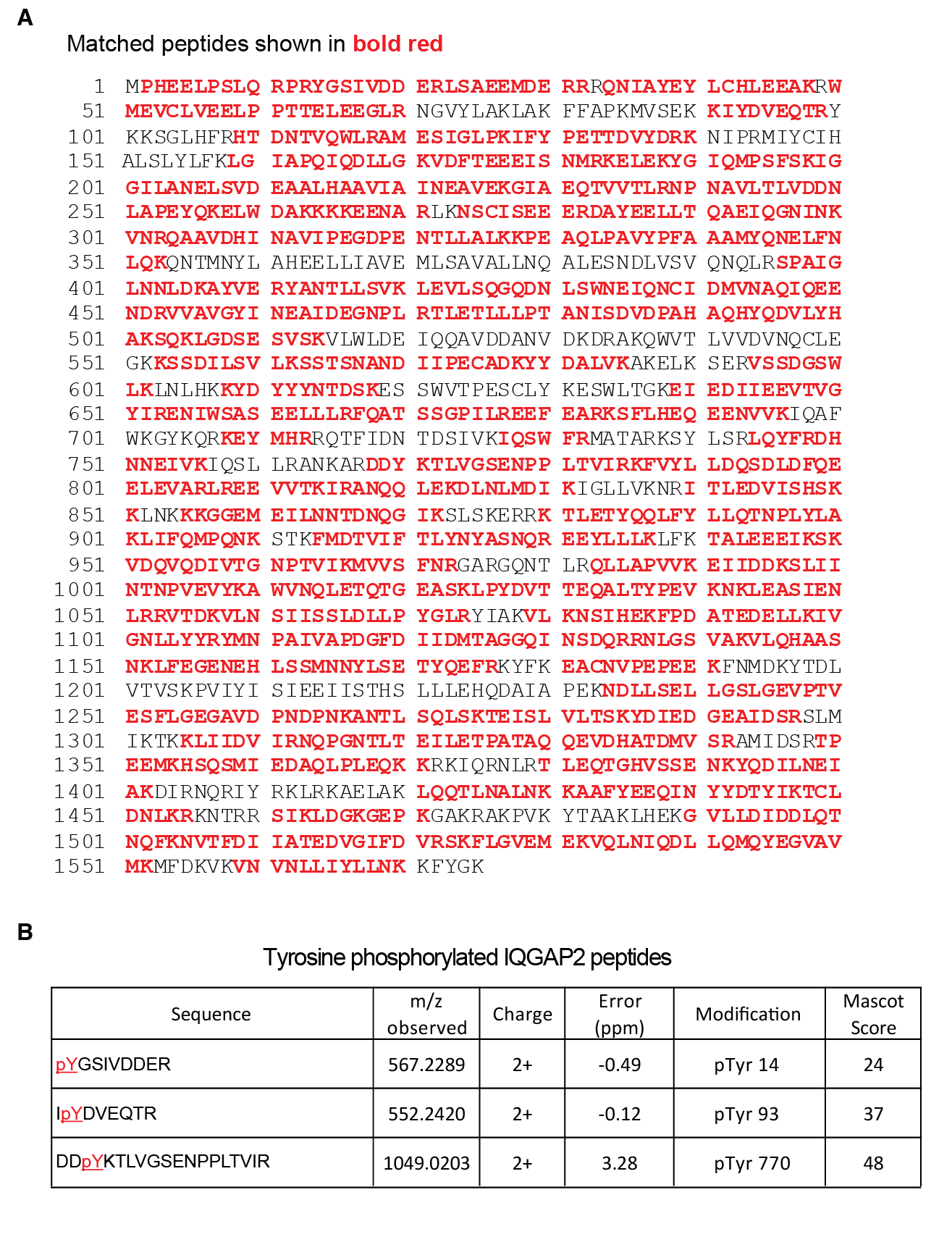


**Figure S4. Summary of IQGAP2 phosphorylation in HepG2 cells treated with vanadate.**

(A) Sequence coverage for IQGAP2 immunoprecipitated from HepG2 cells treated with Na3VO4. This is typical of sequence coverage obtained throughout this work. Sequence coverage includes 50/65 tyrosine residues (77%). (B) Table of tyrosine phosphorylated peptides identified on IQGAP2.


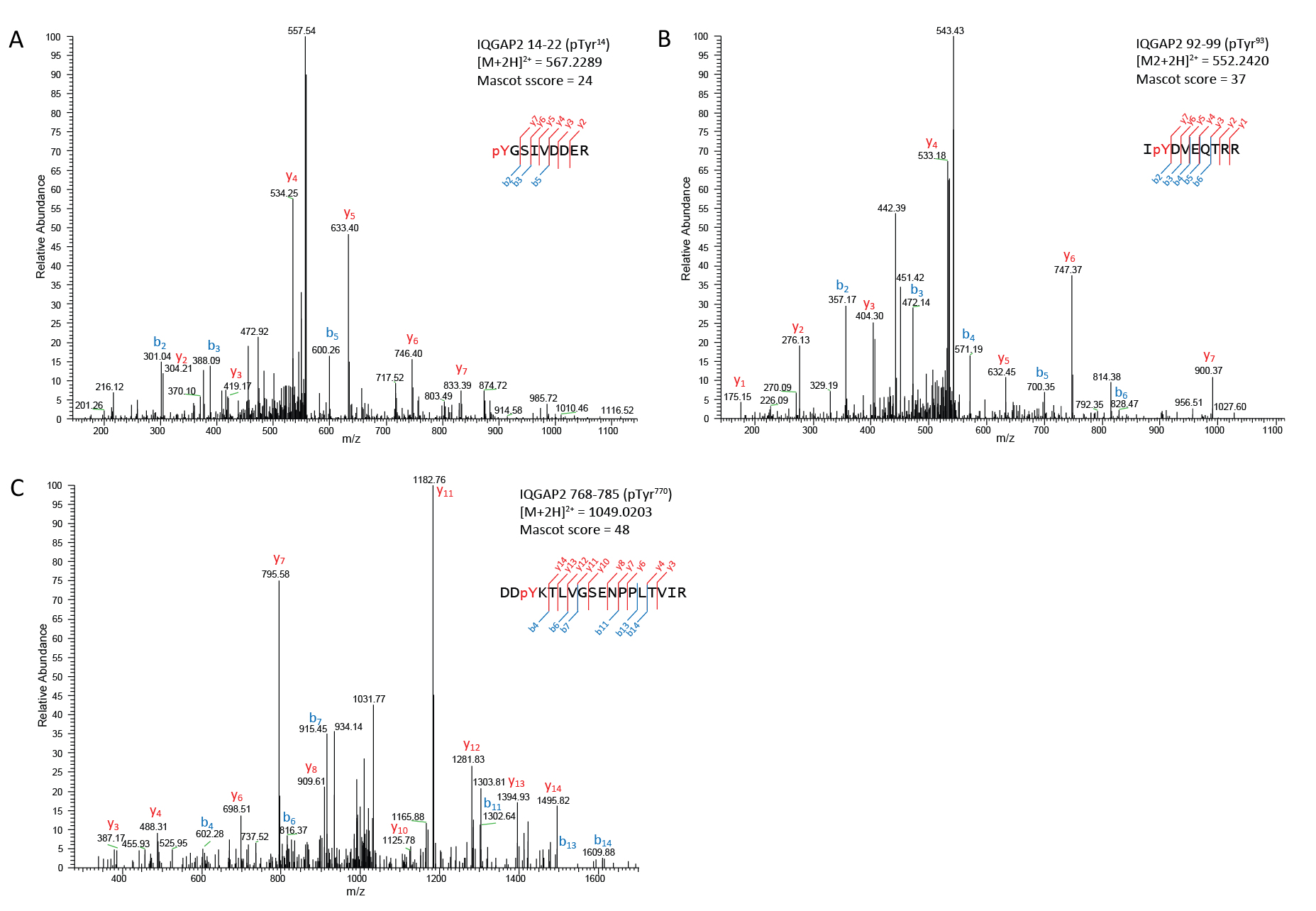


**Figure S5. Mass spectrometric spectra for phosphorylated IQGAP2 peptides in HepG2.**

LC-MS/MS analysis of the in-gel tryptic digested samples allowed the identification of tyrosine phosphorylated residues from HepG2 cells treated with vanadate. A representative MS/MS spectrum for each of the tyrosine phosphorylated peptides are shown for the following regions of IQGAP2: (A) amino acids 14-22 containing phosphorylated Tyr-14, (B) amino acids 92-99 containing phosphorylated Tyr-93, (C) amino acids 768-785 containing phosphorylated Tyr-770.


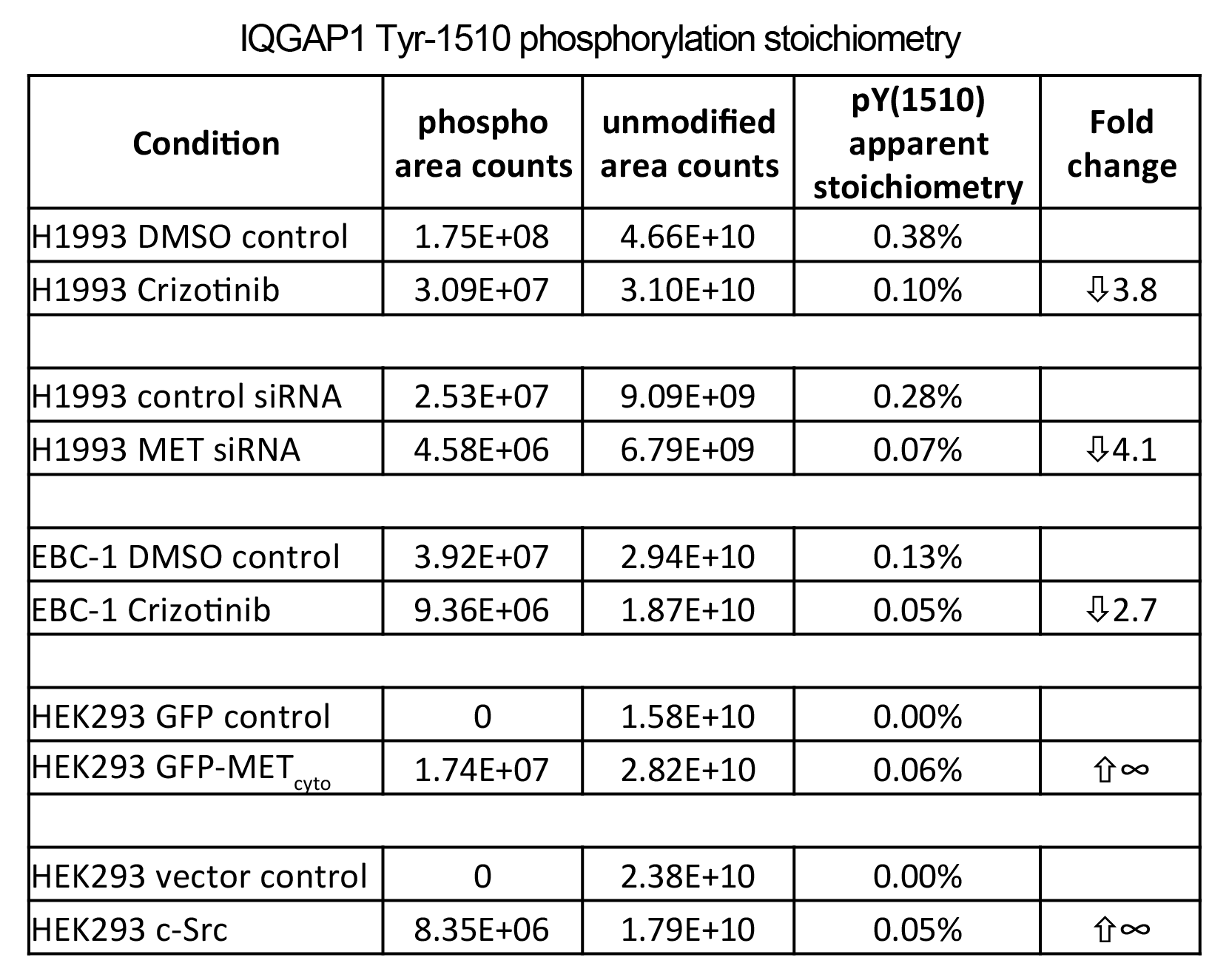


**Figure S6. Summary of IQGAP1 Tyr 1510 phosphorylation under experimental conditions.**

Summary of mass spectrometry quantification of cellular MET-dependent phosphorylation of IQGAP1 Tyr-1510 studied under various pharmacologic and genetic manipulations. Apparent pY(1510) stoichiometry for each condition was calculated as the ratio of extracted ion chromatogram area count abundance of the phosphorylated peptide, divided by the sum total of measured phosphorylated and non-phosphorylated peptide abundances, expressed as percentage (pY1510/(pY1510 + Y1510)*100).
